# Enhanced Anti‐Wetting Methods of Hydrophobic Membrane for Membrane Distillation

**DOI:** 10.1002/advs.202300598

**Published:** 2023-05-23

**Authors:** Honglong Zhang, Xuan Zhao

**Affiliations:** ^1^ Lab of Environmental Science & Technology INET Tsinghua University Beijing 100084 P. R. China

**Keywords:** anti‐wetting, fluorination, hydrophobic membranes, membrane distillation, reentrant‐like structures

## Abstract

Increasing issues of hydrophobic membrane wetting occur in the membrane distillation (MD) process, stimulating the research on enhanced anti‐wetting methods for membrane materials. In recent years, surface structural construction (i.e., constructing reentrant‐like structures), surface chemical modification (i.e., coating organofluorides), and their combination have significantly improved the anti‐wetting properties of the hydrophobic membranes. Besides, these methods change the MD performance (i.e., increased/decreased vapor flux and increased salt rejection). This review first introduces the characterization parameters of wettability and the fundamental principles of membrane surface wetting. Then it summarizes the enhanced anti‐wetting methods, the related principles, and most importantly, the anti‐wetting properties of the resultant membranes. Next, the MD performance of hydrophobic membranes prepared by different enhanced anti‐wetting methods is discussed in desalinating different feeds. Finally, facile and reproducible strategies are aspired for the robust MD membrane in the future.

## Graphical abstract

Frequent wetting issues of hydrophobic membranes in the membrane distillation (MD) process spur the development of enhanced anti‐wetting methods (i.e., surface structural construction and chemical modification) for the MD membrane. The improvement of membrane hydrophobicity and the MD application potentials and challenges of the resultant membranes after implementing these methods are systematically reviewed and discussed.

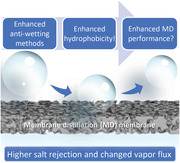

## 1 Introduction

As emerged in 1963, membrane distillation (MD) is a thermally‐driven separation process where only vapor molecules transfer through a microporous hydrophobic membrane under the ideal state.^[^ ^1^ ^]^ The driving force in the MD process is the vapor pressure difference induced by the temperature difference.^[^ ^2^ ^]^ MD has been used for desalinating various feeds, such as seawater, reverse osmosis (RO)/multi‐effect distillation (MED)/multistage flash (MSF) brine, and mine/gas‐produced wastewater.^[^ ^3^ ^]^ The MD performance is directly affected by the structural and physicochemical parameters of the utilized membranes.^[^ ^4^ ^]^ Therefore, one of the most crucial aspects is to have an MD membrane with well‐controlled properties.^[^ ^4^ ^]^ The membranes used in the MD process should be porous and hydrophobic and also exhibit suitable thermal stability and anti‐fouling properties.^[^ ^5^ ^]^

**Figure 1:**
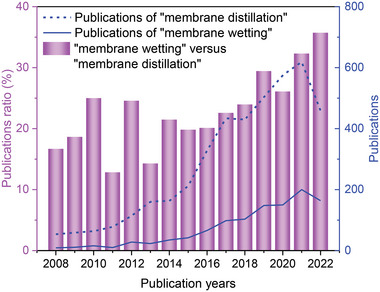
The publications ratio of “membrane wetting” versus “membrane distillation” and the publications of “membrane distillation” and “membrane wetting”. Topics: publications of “membrane distillation”: “membrane distillation” and “water treatment”; publications of “membrane wetting”: “membrane distillation” and “membrane wetting”. Data were from the Web of Science.

Membrane wetting is a vital issue in the MD systems since it induces the decline of permeate flux and quality during the operation.^[^ ^6^ ^]^ The number of publications on MD membrane wetting and the publications ratio of “membrane wetting” versus “membrane distillation” have increased during the past years (**Figure** **1**), suggesting that wettability has gradually become a hot issue of membrane distillation.

Increasing demands for hydrophobic membranes accelerate the development of various enhancing anti‐wetting methods, such as constructing reentrant‐like structures (CRlS), coating organofluorides (COf), and combining the two methods (CRlS‐COf). These methods increased water contact angle (WCA) directly and/or indirectly (e.g., improving WCA by reducing the surface free energy of the membrane via COf), and enhanced the anti‐wetting properties of the MD membrane. In this review, we first introduced the characterization parameters of the membrane wettability. Then we summarized the methods (i.e., CRlS, COf, and CRlS‐COf) to enhance the anti‐wetting properties of the membrane material. Third, we discussed the performance and application potentials of the enhanced hydrophobic membranes in the MD process compared with the pristine ones.

## 2 Fundamental Parameters on Anti‐Wetting Properties

### 2.1 Contact Angle (CA)

Generally, the apparent CA of a liquid droplet (typically a water droplet) on the membrane surface is used to characterize the wettability.^[^ ^7^ ^]^ The apparent CA value of 90° corresponds to the boundary of hydrophilicity and hydrophobicity.^[^ ^8^ ^]^ The Wenzel model^[^ ^9^ ^]^ and the Cassie–Baxter model^[^ ^10^ ^]^ for the apparent CA are commonly used to simulate the wetting state. The Wenzel model is suitable for a complete wetting surface with low and moderate roughness and surface‐free energy (SFE). In contrast, the Cassie‐Baxter model is more likely to be applied to rougher and lower SFE hydrophobic surfaces.

Dynamic CAs (i.e., advancing angle, receding angle, and sliding (roll‐off) angle (*θ_SA_*)) represent the adhesion interaction between the liquid droplet and the membrane.^[^ ^11^ ^]^ Among these, the sliding angle (*θ_SA_*) is the minimum tilting angle causing a droplet to slide off the solid substrate using the tilting‐plate method.^[^ ^12^ ^]^ WCA and *θ_SA_* are used to characterize the water‐repellency of surfaces.^[^ ^13^ ^]^ Surfaces with high WCA (>150°) and low sliding angle (*θ_SA_* < 10°) are superhydrophobic.^[^ ^14^ ^]^ Typically, the WCA (WCA refers to the apparent static CA in this paper) of the membrane prepared by phase inversion counts at a wide range of 80°–160°.^[^ ^15^ ^]^ The recommended WCA is >105° for hydrophobic membranes.^[^ ^15^, ^16^ ^]^

### 2.2 Liquid Entry Pressure (LEP)

Liquid entry pressure (LEP) is an additional parameter of membrane wetting. LEP represents the minimum hydrostatic pressure difference for the liquid to enter the membrane pores.^[^ ^17^ ^]^ LEP mainly combines with the transmembrane pressure difference (Δ*P*), to judge whether membrane pores are readily wetted. If Δ*P* > LEP, the liquid will penetrate the membrane pores and cause membrane wetting.^[^ ^12^, ^18^ ^]^ The thicker the membrane and the smaller its pore size are, the larger the LEP of the membrane will be.^[^ ^19^ ^]^ The LEP of membranes prepared by phase inversion ranges from 50 to 460 kPa,^[^ ^15^ ^]^ with the recommended value being >250 kPa.^[^ ^15^, ^16^, ^20^ ^]^

### 2.3 Surface Free Energy (SFE)

According to Section 2.1, a WCA threshold of 90° is usually used to determine the hydrophobicity or hydrophilicity of the membrane. However, air pockets in the nano‐ and/or micro‐scaled surface may break the continuous solid/liquid interface, allowing the WCA limit to drop to 65°.^[^ ^21^ ^]^ A simplistic definition of hydrophobicity based on WCA may not be relevant in many cases.^[^ ^12^, ^22^ ^]^ Generally, SFE retrieves this discrepancy. SFE is a physical parameter characterizing the intermolecular interactions at an interface, closely relating to the wettability of a solid surface.^[^ ^23^ ^]^ Some researchers believe that supplemental SFE data can provide a complete understanding of membrane‐wetting behavior.^[^ ^12^, ^24^ ^]^

## 3 Methods to Enhance Anti‐Wetting Properties

Different feeds to be desalinated (i.e., seawater, RO/MED/MSF brine, and mine/gas‐produced wastewater) contain various components. Hence, the equipped hydrophobic membranes for MD treatment are also different. For example, the pristine hydrophobic membranes (prepared by phase inversion using commercial plastics of polyvinylidene fluoride (PVDF), polytetrafluoroethylene (PTFE), and polypropylene (PP)^[^ ^25^ ^]^) are competent to desalinate the feed containing soluble salts (e.g., NaCl). But some tricky components (e.g., surfactant or organics with low surface tension), could readily lead to the surface wetting of these pristine membranes. Consequently, enhanced hydrophobic membranes with high WCA and/or LEP and low SFE are needed. Various enhancing methods emerged to achieve the goals, which could be categorized as i) constructing reentrant‐like structures (CRlS), ii) coating organofluorides (COf), and iii) combining the two methods (CRlS‐COf). CRlS maintains air pockets in the membrane surface, while COf reduces the SFE of the membrane surface. Both could help the membrane sustain a Cassie‐Baxter state, producing a superhydrophobic or omniphobic surface. The increasing application of MD in sophisticated water treatment requires the development of methods to enhance the MD membrane hydrophobicity. In this Section, the effects of membrane materials on the wetting parameters are reviewed, and then the processes and principles of methods to enhance the membrane hydrophobicity are introduced.

### 3.1 Effects of Membrane Materials

**Figure 2:**
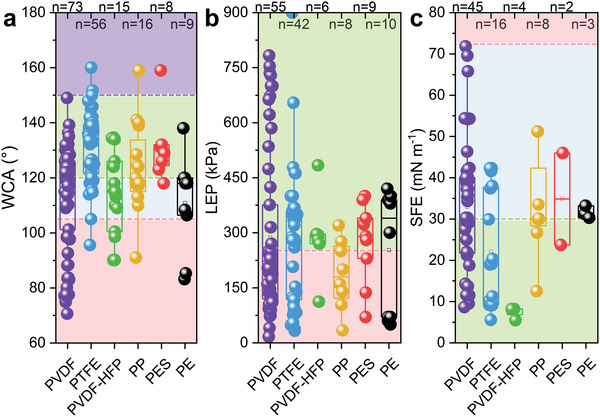
a) WCA, b) LEP, and c) SFE values of commercial (i.e., pristine) hydrophobic membranes used by different materials (n is the sample number). The red dashed lines show a) the recommended WCA value (105°), b) the recommended LEP value (250 kPa, also being the average LEP value of these pristine membranes), and c) the surface tension of water (≈72 mN m^−1^),^[^ ^28^ ^]^ respectively. The green dashed lines indicate the average value of a) WCA and c) SFE of the pristine membranes. The purple dashed line from a) is the WCA value symbolized by superhydrophobic (150°). Raw data are listed in Table S1 (Supporting Information). One‐way analysis of variance (ANOVA) is displayed in Table S2 (Supporting Information).

Apart from well‐hydrophobicity (WCA > 105° and LEP > 250 kPa), other properties (i.e., high membrane porosity (> 75%), proper pore diameter (< 0.3 µm), low tortuosity (1.1–1.2), low thermal conductivity (0.04–0.06 W·m^−1^·K^−1^), and high mechanical strength) of the hydrophobic membrane are recommended for the MD operation.^[^ ^12^, ^16^ ^]^ Generally, PVDF, PTFE, PVDF‐*co*‐hexafluoropropylene (PVDF‐HFP), PP, poly(ether sulfone) (PES), and polyethylene (PE) materials are widely applied to fabricate hydrophobic membranes for MD process (Table S1, Supporting Information). Most of these hydrophobic membranes had highly average WCA values (>110°) and lowly average LEP values (185–290 kPa) (Figure 2a,b). The SFE values varied with the material type. The lowest average SFE value was obtained from PVDF‐HFP, followed by PTFE (**Figure** **2**c), consistent with the fluorine content of the polymers.^[^ ^26^ ^]^ Notably, the molecular weight of the polymer also has effects on the SFE value of the membrane. Chen et al.^[^ ^27^ ^]^ prepared three kinds of PVDF membranes with different molecular weights (i.e., Mw = 410, 1548, and 92840/1367 kDa), and proposed that the more considerable the molecular weight is, the higher the SFE value might be.

### 3.2 Constructing Reentrant‐Like Structures

**Figure 3:**
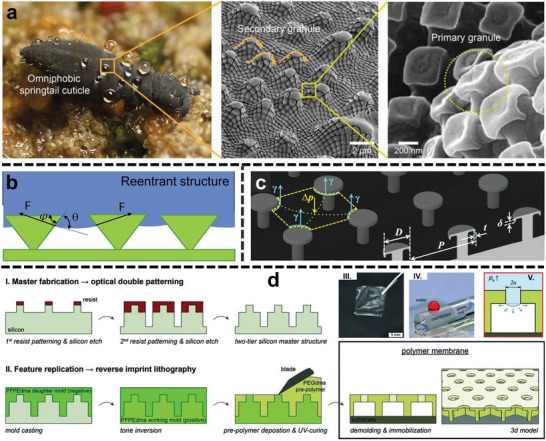
a) Photograph (courtesy of B. Valentine) of a springtail displaying liquid repellency and resistance to high‐pressure raindrops in a flooded habitat (left). SEM images show the hierarchical system in a springtail cuticle composed of primary and secondary granules (middle and right panels). Reproduced with permission.^[^ ^33^ ^]^ Copyright 2018, American Association for the Advancement of Science. b) Possible liquid‐vapor‐solid interfaces on the reentrant structure. Reproduced with permission.^[^ ^7^ ^]^ Copyright 2019, Elsevier. c) The designed surface of micro‐posts with doubly reentrant nano‐overhangs. As key geometric parameters, D is the post‐top diameter, P is the center‐to‐center distance (i.e., pitch) between adjacent posts, and *δ* and *t* are the length and thickness, respectively, of the vertical overhang. Reproduced with permission.^[^ ^36^ ^]^ Copyright 2014, American Association for the Advancement of Science. d) Process scheme for membrane fabrication: I. A two‐tier silicon master structure is fabricated by optical lithography. II. the master structure serves as the template for reverse imprint lithography. Finished membrane demonstration: III. photograph of the springtail‐skin‐inspired polymer membrane. IV. A water droplet (colored with red dye) was deposited on the membrane, which was transferred to a 4‐mm diameter glass rod. V. Schematic side view and optical micrograph series (top view, bright field) of the pressure‐dependent collapse of the plastrons by an expanding waterfront inside the cavities (scale bars: 50 µm). Reproduced with permission.^[^ ^37^ ^]^ Copyright 2014, Wiley‐VCH.

To prevent wetting, the Cassie‐Baxter state of the membrane/liquid interface is necessary for the MD process, and specifically, the air pockets should not be occupied by liquid. A variety of superhydrophobic surfaces (e.g., lotus leaf,^[^ ^29^ ^]^ rice leaf,^[^ ^30^ ^]^ taro leaf,^[^ ^31^ ^]^ butterfly,^[^ ^32^ ^]^ springtail,^[^ ^33^ ^]^ etc.) have been discovered in nature. Studies have shown that the air pockets underneath the water droplet and the low solid/liquid fractional area (i.e., the Cassie–Baxter state) are critical for these superhydrophobic surfaces. Among these, the springtail has received increasing attention due to its remarkable cuticles with intrinsically omniphobic surfaces displaying static repellency of raindrops.^[^ ^33^, ^34^ ^]^ **Figure** **3**a shows the wonderful characteristics of the springtail's cuticle, thanks to the hierarchical reentrant structures with primary mushroom‐shaped granules and secondary grooves‐shaped granules.^[^ ^33^ ^]^ The springtail's cuticle further promoted the modern development of artificial hydrophobic mimics.^[^ ^35^ ^]^

A reentrant structure is a concave topography in which the solid cross‐sectional area decreases from top to bottom.^[^ ^7^ ^]^ As shown in Figure 3b, the net traction F points away from the solid for a reentrant textured surface because the equilibrium contact angle *θ* is more significant than the local texture angle *φ*.^[^ ^7^ ^]^ F opposed to the air pocket, preventing the large contact area between liquid and solid.^[^ ^7^, ^38^ ^]^

However, the preparation of standard reentrant structure shown in Figure 3c refers to complicated processes.^[^ ^36^, ^37^, ^39^ ^]^ For example, Hensel et al.^[^ ^37^ ^]^ fabricated a polymer membrane with a reentrant structure, costing three steps. First, a silicon master structure with two height levels was made by a lithography‐etch‐lithography‐etch approach without any planarization steps for pre‐patterned substrates.^[^ ^37^ ^]^ Second, a perfluoropolyether dimethacrylate (PFPEdma) daughter mold as a negative of the silicon master was cast using a reverse imprint lithography approach.^[^ ^37^ ^]^ The cavities of this working mold were filled with a PEGdma prepolymer solution by doctor‐blade technique without a residual layer on the small pillar structures.^[^ ^37^ ^]^ Finally, the flexible membrane with free‐standing was obtained after demolding (Figure 3d).^[^ ^37^ ^]^ In addition to the template method posed above, the 3D printing technique is also available to construct the standard reentrant structure. The computer‐aided design models were generated with 3D modeling software, and then loaded into the operating software for 3D printing.^[^ ^40^ ^]^ The reentrant structures were printed with the galvo mode for lateral scanning and the piezo mode for vertical scanning.^[^ ^40^ ^]^ The printing platforms were made of fused silica substrates, and the photoresist was mainly composed of 2‐(hydroxymethyl)‐2‐[[(1‐oxoallyl)oxy]methyl]‐1,3‐propanediyl (60–80%).^[^ ^40^ ^]^

Indeed, the template method and 3D printing technique can't prepare the standard reentrant surface, as shown in Figure 3a,c. Moreover, the above fabrication process is too elaborate to prepare the MD membrane on a large scale. In most cases, the surface structure improvement was executed via relatively simple methods (e.g., micromolding phase inversion, electrospinning, attaching nanomaterials, etc.) (**Table** **1**), to fabricate the enhanced hydrophobic membrane with a reentrant‐like structure.

**Table 1:** Constructing reentrant‐like structures (CRlS) methods

Methods	Summary of the CRlS process	Ref.
Micromolding phase inversion	Replicate rough microstructures through the model by casting or embossing.	[41]
	Prepare ultra‐high molecular weight polyethylene microgels by thermally induced phase separation.	[42]
Electrospinning	Construct fiber by electrospinning polymer dope solution.	[43]
	Construct bead‐on‐string fiber through electrospinning by manipulating the electrospinning process parameters.	[44]
Attaching nanomaterials	Attach inorganic nanomaterials^a)^ by covalent bonding or electrostatic attraction.	[45]
	Attach inorganic nanomaterials through the in situ formation by in situ sol‐gel, fastening seeds, or silver mirror reaction.	[38, 43, 45, 46]
	Attach nanomaterials (including inorganic and organic nanomaterials^b)^) by incorporating them into the polymer solution for casting, electrospinning, or electrospray.	[25, 45, 47]
Etching	Etch PVDF/CaCO_3_ membrane through leaching CaCO_3_ by HCl.	[48]

^a)^ inorganic nanomaterials: SiO_2_, ZnO, TiO_2_, etc;

^b)^ organic nanomaterials: PVDF‐HFP, polystyrene, polydimethylsiloxane (PDMS)/PVDF, PDMS‐polyhedral oligomeric silsesquioxane (POSS)/PVDF, etc.

**Figure 4:**
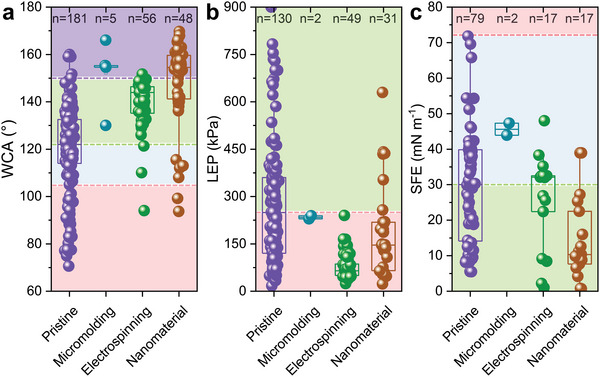
a) WCA, b) LEP, and c) SFE values of pristine hydrophobic membranes and enhanced hydrophobic membranes improved via micromolding phase inversion, electrospinning, and attaching nanomaterials (n is the sample number). The red dashed lines show a) the recommended WCA value (105°), b) the recommended LEP value (250 kPa, also being the average LEP value of these pristine membranes), and c) the surface tension of water (≈72 mN m^−1^),^[^ ^28^ ^]^ respectively. The green dashed lines indicate the average value of a) WCA and c) SFE of the pristine membranes. The purple dashed line from a) is the WCA value symbolized by superhydrophobic (150°). The combination of electrospinning and attaching nanomaterials is categorized as attaching nanomaterials. All membranes involved in this figure are not fluorinated. Raw data are listed in Table S1 (Supporting Information). One‐way ANOVA is displayed in Table S3 (Supporting Information).

**Figure** **4** exhibited the WCA, LEP, and SFE values gaps between the pristine and CRlS membranes. Employing CRlS, the average WCA value of membranes was higher than that of the pristine ones, and partial WCA values exceeded 150°. But for LEP and SFE, the superiority of CRlS was well‐hidden. Moreover, the LEP values of the electrospun nanofiber membranes (ENMs) got lower, which might be attributed to the large pore size of the ENMs. Overall, CRlS significantly improved the WCA level of the hydrophobic membranes and promoted their anti‐wetting properties. Table 1 collected the CRlS methods emerging in recent years. The detailed process and the resultant membrane properties were displayed in Sections 3.2.1–3.2.3.

#### 3.2.1 Micromolding Phase Inversion

The phase inversion method has been widely applied in the preparation of polymer membranes,^[^ ^49^ ^]^ as briefly described as follows: i) a homogeneous solution containing polymer at the desired concentration was prepared, and ii) the thermodynamic state of the casting solution was changed in some way, to cause phase separation of the homogeneous polymer solution. The concentrated polymer phase was continuous, forming the main skeleton of the membrane after curing. The diluted polymer phase was dispersed, forming membrane pores after elution. Non‐solvent‐induced phase separation (NIPS) and vapor‐induced phase separation (VIPS) were the most used phase inversion methods.

**Figure 5:**
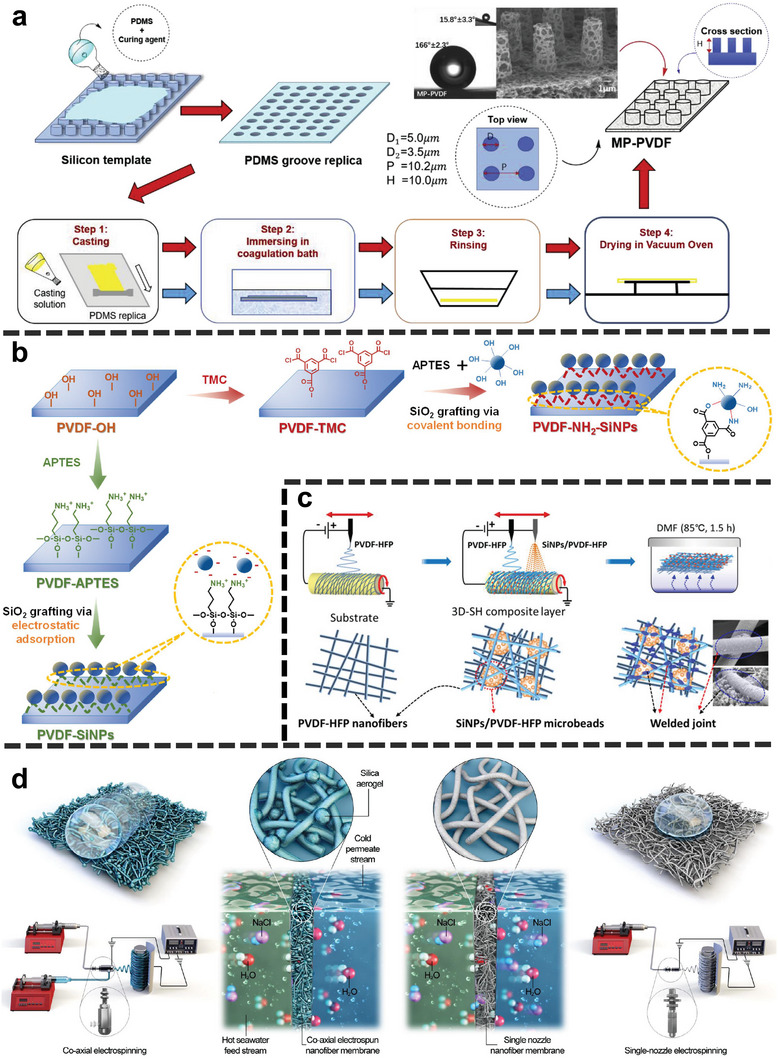
a) Schematic illustration for fabricating micropillar PVDF membranes (MP‐PVDF) by micromolding phase inversion. The silicon wafer mold has pillars with the dimension of 5 µm (diameter), 10 µm (height), and 10 µm (period). Reproduced with permission.^[^ ^41b^ ^]^ Copyright 2019, Elsevier. b) Schematic illustration of the fabrication processes of grafting SiO_2_ nanoparticles (SiNPs) PVDF membranes via covalent bonding (PVDF‐C) and electrostatic attraction (PVDF‐E). Reproduced with permission.^[^ ^45k^ ^]^ Copyright 2022, Elsevier. c) Schematic illustration of the SiNP beads/PVDF‐HFP ENM by electrospinning‐*co*‐electrospray. Reproduced with permission.^[^ ^47c^ ^]^ Copyright 2019, American Chemical Society. d) Schematic illustrations of the co‐axial ENM and the pristine ENM by single‐nozzle electrospinning, and their properties. Reproduced with permission.^[^ ^43k^ ^]^ Copyright 2021, Elsevier.

Micromolding phase inversion generally refers to preparing a membrane with a controllable surface morphology (e.g., reentrant‐like structure) by template method. Typically, a template with a specific shape (e.g., micropillars^[^ ^41b^ ^]^) is essential in micromolding phase inversion.^[^ ^41a–d^ ^]^ **Figure** **5**a displayed a classic process of micromolding phase inversion.^[^ ^41b^ ^]^ To construct the reentrant‐like structure on the membrane, a polydimethylsiloxane (PDMS) daughter mold was prepared first by casting the PDMS solution onto the silicon wafer template.^[^ ^41b^ ^]^ The pre‐mixed PVDF casting solution was spread uniformly on the PDMS replica on top of a glass plate to a thickness of 600 µm using a casting knife.^[^ ^41b^ ^]^ After demolding, the micropillar PVDF membrane (MP‐PVDF) was obtained^[^ ^41b,c^ ^]^ and exhibited excellent anti‐wetting properties considering its high WCA (166.0° ± 2.3°) and low *θ_SA_* (15.8° ± 3.3°). In contrast, the pristine PVDF membrane has a WCA of 139.2° ± 3.7° and *θ_SA_* of >90°.^[^ ^41b^ ^]^

However, the extra tricky fabrication of mold hinders its scale‐up. It had been reported that the micro‐molded phase transition process might be optimized by using ubiquitous stainless‐steel mesh as a template for micromolding phase inversion.^[^ ^50^ ^]^ Moreover, Quan et al.^[^ ^42^ ^]^ explored a template‐free process and prepared a “particle‐like” structure using ultra‐high molecular weight polyethylene. The resultant membrane without fluorination possesses high WCA (154.6°) and low *θ_SA_* (2.5°).^[^ ^42^ ^]^ The template‐free process can reduce the complexity of constructing a micro‐structured surface and is expected to become a promising technology.

#### 3.2.2 Electrospinning

Electrospinning is a promising method to produce ultrafine (in nanometers) fibers by charging and ejecting a polymer melt or solution through a spinneret under a high‐voltage electric field and solidifying or coagulating it to form a filament.^[^ ^51^ ^]^ Because of the cylindrical reentrant‐like structure of nanofibers produced by electrospinning, it can also be called the in situ preparation technique of hydrophobic structure.^[^ ^43^, ^52^ ^]^ Compared to the phase inversion membranes, electrospun nanofiber membranes (ENMs) have unique structural advantages (i.e., high porosity (≈80%), narrow pore size distribution, low tortuosity, etc.).^[^ ^52b^ ^]^ PVDF and PVDF‐HFP powder/particles could readily be dissolved in organic solvents to be electrospun. Unfortunately, PTFE is barely to be electrospun due to its high viscoelastic property.^[^ ^43g^ ^]^ Therefore, additives (e.g., poly(vinylalcohol)^[^ ^53^ ^]^ and poly(ethylene oxide)^[^ ^43h^ ^]^) were added to the PTFE emulsion for the ENM preparation.^[^ ^43g^ ^]^

The fabrication procedure of ENM involves dope solution preparation and ENM fabrication by electrospinning.^[^ ^54^ ^]^ Take a PVDF ENM for instance.^[^ ^43a^ ^]^ The dope solution was prepared by dissolving 10 wt.% PVDF into a mixed solvent of 60 wt.% *N, N*‐dimethylformamide (DMF) and 30 wt.% acetone. Subsequently, the dope solution was loaded into the syringe to electrospun.^[^ ^43a^ ^]^ As displayed in Figure 4a, the WCA of ENM is generally confined to the range of 120°–150° with few outliers, meaning good anti‐wetting properties and stability.

**Figure 6:**
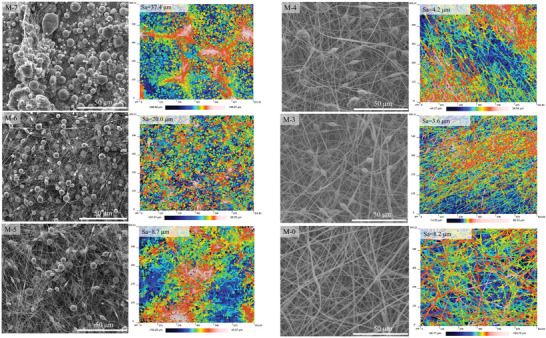
Scanning electron microscopy and optical profiler images of the PVDF electrospun membrane surface by varying the DMAC to acetone mass ratio. Experimental conditions: M‐7–M‐3: PVDF concentration = 17 wt.%, and DMAC/acetone ratio = 7:3 (M‐7), 6:4 (M‐6), 5:5 (M‐5), 4:6 (M‐4), and 3:7 (M‐3); M‐0: PVDF concentration = 25 wt.%, and DMAC/acetone ratio = 7:3. Reproduced with permission.^[^ ^43d^ ^]^ Copyright 2022, Elsevier.

High‐boiling‐point solvents could hinder fiber solidification.^[^ ^44^, ^55^ ^]^ As a result, beaded nanofibers could be fabricated using a high‐boiling‐point solvent during electrospinning.^[^ ^44^, ^55^ ^]^ Interestingly, beaded nanofiber membranes possessed higher WCA than the non‐beaded ones,^[^ ^44^, ^56^ ^]^ which might be ascribed to its hierarchically reentrant‐like structure. Using *N*‐methyl‐2‐pyrrolidone with the high boiling point solvent instead of DMF/acetone, Zhou et al.^[^ ^44^ ^]^ electrospun a beaded nanofiber membrane with superhydrophobicity (WCA > 150°). Remarkably, by the one‐step controlled‐electrospinning method, the resultant membrane possessed a hierarchical reentrant‐like structure. Similarly, Hu et al.^[^ ^43d^ ^]^ used another high‐boiling‐point solvent (*N, N*‐dimethylacetamide (DMAC)) and acetone to dissolve PVDF, and co‐electrospun a series of beaded nanofiber membranes by tailoring the DMAC/acetone ratio (i.e., DMAC: acetone = 7:3, 6:4, 5:5, 4:6, and 3:7). **Figure** **6** presented the variable shaped membranes. By increasing the DMAC/acetone ratio, the classical fiber morphology (M‐0) changed from nanofiber to beads.^[^ ^43d^ ^]^ With the increase in bead density, the WCA of the beaded nanofiber membrane increased, and the WCA value of the M‐7 membrane was the highest, reaching 148°.^[^ ^43d^ ^]^ Besides solvent type, the beaded nanofiber membrane could also be fabricated by controlling other electrospinning process parameters (e.g., applied voltage, polymer concentration, dope solution speed, etc.).^[^ ^55^, ^56^, ^57^ ^]^

Besides, Guo et al.^[^ ^47h^ ^]^ also prepared the beaded nanofiber membrane using electrospinning‐*co*‐electrospray. The nanofibers and nanobeads were formed via electrospinning using 15% PVDF‐HFP dope solution and electrospray using 5% PVDF‐HFP dope solution, respectively.^[^ ^47h^ ^]^ The resultant membrane possessed a superhydrophobic surface (WCA = 153.8° ± 0.19°). This method was also reported to fabricate PVDF‐HFP ENM with polystyrene beads.^[^ ^47i^ ^]^ Still, the two‐step technique is sophisticated for scaling up.^[^ ^56b^ ^]^ Similar beaded nanofiber membranes with hierarchically reentrant‐like structures could also be achieved through nanomaterials attachment on the ENMs. These nanomaterials usually include SiNPs,^[^ ^43^, ^45^, ^58^ ^]^ ZnO nanoparticles/rods,^[^ ^43^, ^47^ ^]^ TiO_2_ nanoparticles/rods,^[^ ^46^, ^59^ ^]^ Ag nanoparticles,^[^ ^46g^ ^]^ etc. (Table S1, Supporting Information). After fluorination, a series of superhydrophobic membranes with WCA > 150° was prepared. The detailed discussion presents in Section 3.2.3.

#### 3.2.3 Attaching Nanomaterials

Attaching nanomaterials to construct surface morphology (i.e., nano or microparticles/rods/beads/flowers) of hydrophobic membranes can be achieved through deposition, coating, electrospray, and incorporation. Generally, these nanomaterials includes SiO_2_,^[^ ^12^, ^43^, ^45^, ^47^, ^58^, ^60^ ^]^ ZnO,^[^ ^38^, ^43^, ^45^, ^46^, ^47^ ^]^ TiO_2_,^[^ ^46c^ ^–^ ^46^, ^47^, ^59^, ^61^ ^]^ Ag,^[^ ^46^, ^62^ ^]^ PVDF‐HFP,^[^ ^47h^ ^]^ polystyrene,^[^ ^47i^ ^]^ PDMS/PVDF,^[^ ^25^, ^47^ ^]^ PDMS‐polyhedral oligomeric silsesquioxane (POSS)/PVDF cauliflower‐shaped,^[^ ^47m^ ^]^ PDMS‐polydopamine,^[^ ^63^ ^]^ etc. In this Section, we introduced the process of attaching SiO_2_ nanoparticles (SiNPs) due to their widespread applications and relatively low cost.^[^ ^64^ ^]^

The in situ sol‐gel method could coat SiNPs on the membrane surface, using a precursor of SiNPs named tetraethyl orthosilicate (TEOS).^[^ ^45a,b^ ^]^ For instance, the surface of the pristine PP membrane was hydroxylated (e.g., UV/persulfate treatment) first, to provide the —OH active sites for SiNPs binding.^[^ ^45b^ ^]^ Next, the hydroxylated PP membrane (PP—OH) was soaked in the mixture solution of ethanol and NH_3_·H_2_O, and TEOS was dropwise added into the mixture.^[^ ^45a,b^ ^]^ During the process, SiNPs were in situ formed and bound with the hydroxyl group on the PP—OH surface.^[^ ^45b^ ^]^ The resultant membrane was washed with ethanol three times and treated at 60 °C for 3 h to complete the gelation process and obtain a relatively stable SiNPs coating.^[^ ^45b^ ^]^ The reentrant‐like structure was successfully constructed by attaching SiNPs. However, fluorination became indispensable owing to the hydrophilicity of SiNPs. Shao et al.^[^ ^45a^ ^]^ examined the effects of fluorination on the WCA of the PP membranes attaching SiNPs (SiNPs‐PP). The WCA (60.5°) of SiNPs‐PP was lower than that of the pristine one (118°). After fluorination, the final PP membrane (F‐SiNPs‐PP) had a WCA value of 159° and was superhydrophobic.^[^ ^45a^ ^]^ In the process, attaching SiNPs and fluorination increased the WCA of the pristine PP membrane by 41°. However, their relative contribution to hydrophobic enhancement remains unknown and requires further investigation.

In addition to the in situ sol‐gel method, the grafting approach via covalent bonding or electrostatic attraction^[^ ^45k^ ^–^ ^m^ ^]^ was illustrated in Figure 5b. Pre‐hydroxylation of the pristine membrane was also required to offer the —OH active sites for the additives. Unlike UV/persulfate treatment in PP hydroxylation,^[^ ^45b^ ^]^ alkaline treatment was applied to hydroxylate PVDF.^[^ ^45k^ ^]^ Defluorination and oxygenation of PVDF polymer chains occurred during the alkaline treatment, and fluorine was substituted with hydroxyl groups.^[^ ^65^ ^]^ Afterward, the PVDF‐OH (hydroxylate PVDF membrane) was grafted with SiNPs by covalent bonding or electrostatic attraction. In the covalent bonding pathway, trimesoyl chloride (TMC) was added and bound with the —OH site of PVDF‐OH, leading to a surface with abundant acid chloride groups (PVDF‐TMC).^[^ ^45k^ ^]^ Subsequently, the obtained membrane was immersed in acetone containing 1.0% (w/v) SiNPs functionalized by (3‐aminopropyl) triethoxysilane (APTES) (NH_2_‐SiNPs) for 2 h under gentle shaking.^[^ ^45k^ ^]^ Finally, covalently grafting between amino/hydroxyl groups of NH_2_‐SiNPs and acyl chlorides of TMC occurred on the PVDF‐TMC surface.^[^ ^45k^ ^]^ In the electrostatic attraction pathway, APTES covalently reacted with the hydroxyl groups on the PVDF‐OH surface, to form a silylated PVDF membrane (PVDF‐APTES) with positive charges.^[^ ^45k^ ^]^ The negatively charged SiNPs were electrostatically attracted to the PVDF‐APTES surface.^[^ ^45m^ ^]^

The membranes (PVDF‐NH_2_‐SiNPs) obtained by the two pathways generally require fluorination and are denoted as PVDF—C (covalent bonding) and PVDF‐E (electrostatic attraction), respectively. Compared with the pristine membrane (WCA = 119.8° ± 1.5°), both PVDF‐C and PVDF‐E possess high WCA. The anti‐wetting properties of PVDF‐C (WCA = 166.5° ± 1.4°, *θ_SA_* = 5.0° ± 1.1°) are more significant than that of PVDF‐E.^[^ ^45k^ ^]^

A combination of micromolding phase inversion and attaching nanomaterials would generate the hierarchical reentrant‐like structure. Xiao et al.^[^ ^45q^ ^]^ deposited SiNPs on the MP‐PVDF surface by the following process. The MP‐PVDF was treated with O_2_ plasma for hydroxylation and then charged positively by introducing APTES.^[^ ^45q^ ^]^ Subsequently, the resultant membrane was soaked into the aqueous SiNP suspension prepared by the Stöber process.^[^ ^45^, ^66^ ^]^ Based on electrostatic interaction, the negatively charged SiNPs were absorbed on the membrane surface.^[^ ^45q^ ^]^ The formed SiNPs‐MP‐PVDF had a high WCA of 175.6° ± 2.1° after fluorination and displayed more extraordinary anti‐wetting properties than that of MP‐PVDF (WCA = 0155.3° ± 1.7°).^[^ ^45q^ ^]^

Furthermore, a SiNPs‐macro‐corrugated membrane with three hierarchical levels of reentrant‐like structure (i.e., first level: macro corrugations, second level: micro PVDF spherulites, and third level: SiNPs) was designed and fabricated by Kharraz et al.^[^ ^67^ ^]^ The macro‐corrugated membrane comprised two layers and was forged through a combination of NIPS and VIPS for the first and second layers, respectively.^[^ ^67^ ^]^ Among them, the second layer made by VIPS was related to micromolding phase inversion, constructing a macro‐corrugated reentrant‐like structure.^[^ ^67^ ^]^ After alkaline treatment and NH_2_‐functionalization, the positively charged macro‐corrugated membrane could attract the negatively charged SiNPs, to produce the SiNPs‐macro‐corrugated membrane.^[^ ^67^ ^]^ After fluorination, its WCA and OCA (oil CA) reach 160.8° ± 2.3° and 154.3° ± 1.9°, respectively.^[^ ^67^ ^]^ Liao et al.^[^ ^68^ ^]^ prepared a millimeter‐scaled corrugated (parallel and perpendicular modes) membrane covered with nanometer‐scaled SiNPs. The authors found that the perpendicular corrugation mode featured the best scaling resistance owing to the turbulent effect induced by the perpendicular feed flow on the corrugated patterns and the consequent alleviation of concentration polarization.^[^ ^68^ ^]^

As introduced in Section 3.2.2, another method of constructing a hierarchical reentrant‐like structure is to attach nanomaterials to the ENMs (Figure 5c,d). Electrospinning combined with electrospraying is a common method. For instance, the PVDF‐HFP electrospinning dope and SiNPs/PVDF‐HFP electrospraying dope were applied to form the membrane.^[^ ^47c^ ^]^ The obtained PVDF‐HFP ENM with SiNPs/PVDF‐HFP microbeads (Figure 5c) possessed a WCA higher than 160° after fluorination.^[^ ^47c^ ^]^ Another similar method is co‐axial electrospinning, as shown in Figure 5d.^[^ ^43k^ ^]^ Before electrospinning, the SiO_2_ aerogel/PVDF‐HFP dope (i.e., the sheath solution) was mixed in the PVDF‐HFP core dope through a co‐axial adaptor.^[^ ^43k^ ^]^ Fluorination‐free was achieved, since the SiO_2_ aerogel was coated by PVDF‐HFP.^[^ ^43k^ ^]^ The WCA of the resulting membrane reaches 169.7° ± 0.7°.^[^ ^43k^ ^]^

### 3.3 Coating Organofluorides

The SFE values of pristine membranes are generally higher than that of these modified (especially surface chemical modification), as presented in Figure S1 (Supporting Information). After fluorination, the SFE values of the membrane could be lower than 1 mN m^−1^. Besides, silanization also could reduce the SFE values, albeit limited. This trend can be ascribed to the low SFE of organofluorides and organosilanes, especially organofluorides.^[^ ^45r^ ^]^ In general, merely changing the surface structure (e.g., attaching nanoparticles) can't significantly reduce the SFE of the membrane. In the absence of fluorination and/or silanization, the attachment of nanoparticles (e.g., SiNPs) even increases the SFE value of the membrane, due to the abundance of hydroxyl groups on the surface of SiNPs.^[^ ^69^ ^]^ To address this issue, Toh et al.^[^ ^60b^ ^]^ grafted polydimethylsilane on the SiNPs surface (silanization for SiNPs) before its attachment to the membrane, which eliminated the adverse effects of the hydroxyl group and incidentally reduced SFE. Lu et al.^[^ ^45o^ ^]^ increased the SFE value of PVDF hollow‐fiber membrane from 18.8 mN m^−1^ to 33.08 mN m^−1^ via depositing SiNPs on the membrane surface, then decreased it to 14.0 mN m^−1^ by a Teflon AF 2400 (an organofluoride) coating. Therefore, in many cases, attaching nanomaterial must be combined with fluorination to reduce the SFE of the membrane (Section 3.2.3). The most common fluorination approach is coating organofluorides (COf). It should be noted that the mentioned “coating” commonly means “coating on the membrane surface”, and sometimes refers to “coating on the attached nanomaterials (e.g., SiNPs)” (Table S1, Supporting Information). In addition, CF_4_ plasma treatment^[^ ^41^, ^70^ ^]^ and incorporating organofluorides (e.g., chlorotrifluoroethylene) into the casting/dope solution^[^ ^41^, ^71^ ^]^ can also fluorinate membrane surface, which is not discussed in this paper due to their infrequent use.

**Figure 7:**
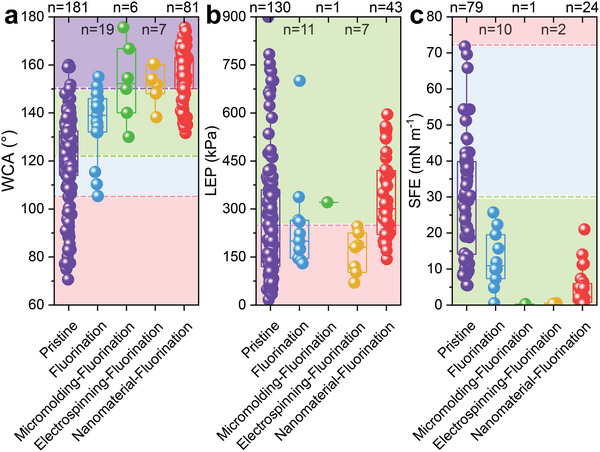
a) WCA, b) LEP, and c) SFE values of pristine hydrophobic membranes and enhanced hydrophobic membranes improved via fluorination, micromolding phase inversion‐fluorination, electrospinning‐fluorination, and attaching nanomaterials‐fluorination (n is the sample number). The red dashed lines show a) the recommended WCA value (105°), b) the recommended LEP value (250 kPa, also being the average LEP value of these pristine membranes), and c) the surface tension of water (≈72 mN m^−1^),^[^ ^28^ ^]^ respectively. The green dashed lines indicate the average value of a) WCA and c) SFE of the pristine membranes. The purple dashed line from a) is the WCA value symbolized by superhydrophobic (150°). The combination of micromolding phase inversion/electrospinning and attaching nanomaterials is categorized as attaching nanomaterials. Raw data are listed in Table S1 (Supporting Information). One‐way ANOVA is displayed in Table S4 (Supporting Information).

Figure 7 revealed the crucial role of fluorination in optimizing the SFE and WCA of MD membranes. Notably, all membranes modified by CRlS‐COf were elevated to a superhydrophobic level (**Figure** **7**). After fluorination, the structure of the membrane surface was hardly affected, supporting that the surface chemistry (i.e., SFE) had been strongly modified.^[^ ^72^ ^]^ Besides, fluorination increased the LEP values of all CRlS membranes by comparing Figure 4 and Figure 7. After fluorination, the average LEP values of membranes modified through micromolding phase inversion or attaching nanomaterials exceeded 250 kPa.

**Figure 8:**
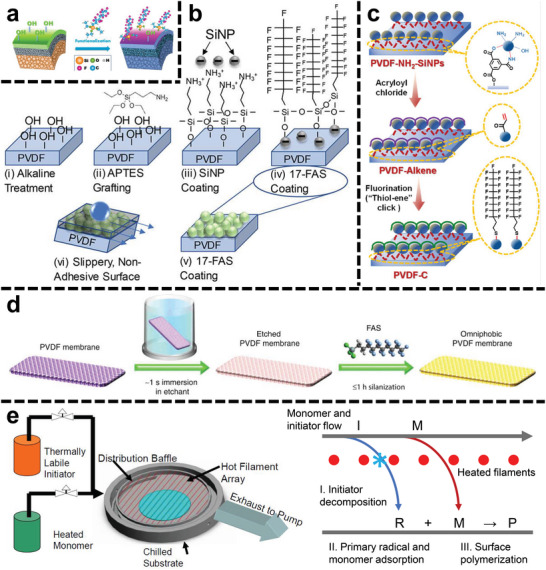
Schematic illustration for the hydrolysis‐condensation reaction between the perfluoroalkylsilanes and a) the hydroxyl groups on the ceramic membrane surface^[^ ^73^ ^]^ and b) the SiNPs surface.^[^ ^45m^ ^]^ Reproduced with permission.^[^ ^45^, ^73^ ^]^ Copyright 2017, American Chemical Society; Copyright 2018, American Chemical Society. c) Schematic illustration for the thiol‐ene click reaction between 1H,1H,2H,2H‐perfluorodecanethiol and the alkene on the PVDF‐Alkene. Reproduced with permission.^[^ ^45k^ ^]^ Copyright 2022, Elsevier. d) Schematic illustration for the hydrolysis‐condensation reaction between the perfluoroalkylsilanes and the etched membrane. Reproduced with permission.^[^ ^74^ ^]^ Copyright 2019, Nature Publishing Group. e) Schematic illustration of initiated chemical vapor deposition (iCVD) equipment (left) and general deposition mechanism (right). Reproduced with permission.^[^ ^76^ ^]^ Copyright 2011, American Chemical Society.

Commonly, the abundant sites (i.e., oxygen‐containing groups) are necessitated for organofluorides (e.g., perfluoroalkylsilanes) being bound on the membrane surface via the hydrolysis‐condensation reaction (**Figure** **8**a).^[^ ^45^, ^73^ ^]^ When SiNPs have been attached for CRlS, the perfluoroalkylsilanes could bind with the hydroxyl groups of SiNPs’ surface without pre‐hydroxylation (Figure 8b).^[^ ^45m^ ^]^ Besides the hydrolysis–condensation reaction, Li et al.^[^ ^45k^ ^]^ proposed another approach based on covalent bonding. The authors grafted acryloyl chloride onto the modified membrane (i.e., PVDF‐NH_2_‐SiNPs in Figures 5b,8c), and obtained the alkene‐functionalized membrane (PVDF‐Alkene).^[^ ^45k^ ^]^ Afterward, the fluorinated membrane was achieved via a thiol‐ene click reaction on the PVDF‐Alkene surface (Figure 8c).^[^ ^45k^ ^]^ Wang et al.^[^ ^74^ ^]^ explored an ultrafast pretreatment technology. A sodium/naphthalene‐based etching solution stripped the fluorine from the backbone of PVDF.^[^ ^74^ ^]^ Meanwhile, oxygen‐containing groups (i.e., hydroxyl and carboxyl groups) were created to provide active sites for the subsequent grafting of the perfluoroalkylsilanes (Figure 8d).^[^ ^74^ ^]^ The whole etching process cost ≈1 s, without significant change of membrane morphology.^[^ ^74^ ^]^

Interestingly, a non‐covalent bond process was reported by Wu et al.^[^ ^43i^ ^]^ Because of its low polarity, the long‐chain perfluoroalkylsilanes (i.e., 1H, 1H, 2H, 2H‐perfluorodecyltrichlorosilane (FDTS)) vapor molecules physically adsorbed on the hydrophobic membrane surface during the vapor deposition.^[^ ^43^, ^75^ ^]^ At the same time, the highly moisture‐sensitive trichlorosilane head was exposed and easily hydrolyzed via trace moisture in the air.^[^ ^43^, ^75^ ^]^ Subsequently, intermolecular polycondensation could occur among the hydrolyzed FDTS molecules to form polysiloxane networks with a long fluoroalkyl chain exposed on the surface.^[^ ^43i^ ^]^ Moreover, the water molecules produced by polycondensation induced the hydrolysis of other FDTS molecules, thus facilitating the deposition process throughout the membrane surface.^[^ ^43i^ ^]^

Chemical vapor deposition (CVD) is an emerging, facile, scalable approach to coat organofluorides.^[^ ^77^ ^]^ The used configuration is initiated chemical vapor deposition (iCVD), whose equipment and fluorination mechanism are shown in Figure 8e. Gleason's group used 1H,1H,2H,2H‐perfluorodecyl acrylate (PFDA) and tert‐butyl peroxide (TBPO) as the fluorinated monomer and initiator, respectively.^[^ ^76^ ^]^ A temperature of ≈250 °C from the filaments resulted in the selective formation of free radicals from TBPO without the decomposition of PFDA. Then adsorption and polymerization were performed on a cooled substrate to form a fluorinated poly(1H,1H,2H,2H‐perfluorodecyl acrylate) (pPFDA) deposition on the membrane surface.^[^ ^76^ ^]^

Notably, the bioaccumulation potentials of organofluorides should not be ignored.^[^ ^78^ ^]^ Gharabli et al.^[^ ^78a^ ^]^ implemented the EPI Suite software of the U.S. Environmental Protection Agency to estimate the bioaccumulation potentials of the used organofluorides in COf. The organofluorides were identified as bioaccumulative when their log BAF, log BCF, or log K_ow_ values were higher than 3.7, 3, or 5, accordingly,^[^ ^78^, ^79^ ^]^ as summarized in Table S5 (Supporting Information). Since the long‐chain organofluorides tend to accumulate in animals through food chains,^[^ ^78^, ^80^ ^]^ as reflected by the high value of log K_ow_,^[^ ^78a^ ^]^ some ultrashort‐chain organofluorides (e.g., FC1 in Table S5, Supporting Information) or other green modifiers, and even the modifier‐free methods, should be considered more in practice.^[^ ^43^, ^44^, ^45^, ^47^, ^78^, ^81^ ^]^

## 4 MD Performance of Enhanced Hydrophobic Membranes

The methods, including CRlS, COf, and CRlS‐COf, have efficiently improved the WCA of the hydrophobic membrane and its anti‐wetting properties. To understand the relationship between the anti‐wetting properties and the MD performance, we evaluated the flux and conductivity/salt rejection of these enhanced hydrophobic membranes in the MD process compared to the pristine ones. In addition, the application potentials of the enhanced hydrophobic membranes in water treatment were proposed rationally.

Table S6 (Supporting Information) shows that the reported feeds are salty waters of NaCl. Researchers prefer to add surfactants with low surface tension routinely (e.g., sodium dodecyl sulfate (SDS)) into the salty feeds to highlight the excellent anti‐wetting properties of the enhanced MD membranes over the pristine ones. Artificial feeds were classified as follows: salty feed (NaCl), gypsum feed (CaSO_4_ or Na_2_SO_4_/CaCl_2_), organic brine (NaCl and organics), surfactant brine (NaCl and surfactants), surfactants organic brine (NaCl, organics, and surfactants), oily brine (NaCl and oil), oil‐in‐water emulsion (NaCl, surfactants, and oil), etc., and had been used to test the MD performance of hydrophobic membranes. A minority of studies utilized real water and its mimics (i.e., seawater,^[^ ^41^, ^43^, ^82^ ^]^ simulated dyeing wastewater,^[^ ^42^ ^]^ biologically pre‐treated coking wastewater,^[^ ^45j^ ^]^ synthetic shale gas wastewater,^[^ ^45k^ ^]^ and imitative oil/gas production waste emulsion^[^ ^45s,t^ ^]^) as the feed solution for the MD test of the enhanced hydrophobic membranes.

### 4.1 Salty Feed

**Figure 9:**
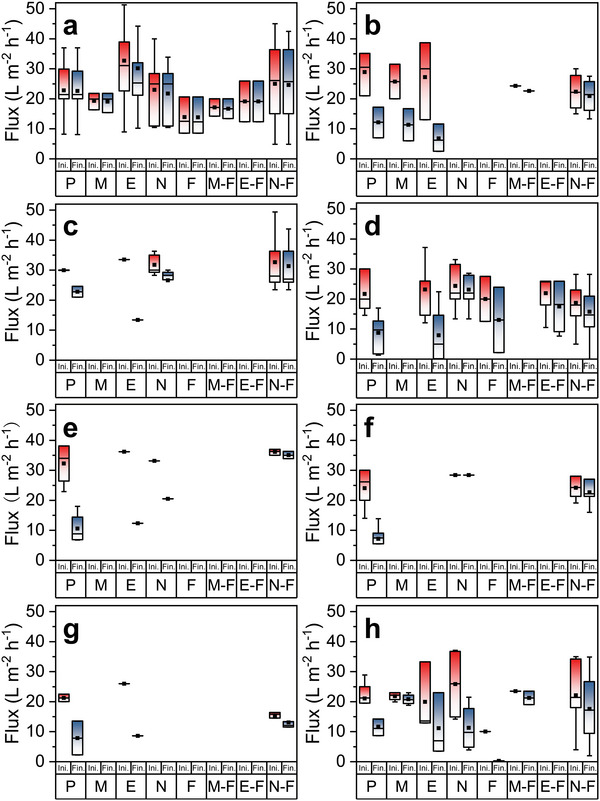
The initial and final permeate flux of MD equipped with the pristine (P), micromolding phase inversion (M), electrospinning (E), attaching nanomaterials (N), fluorinated (F), micromolding phase inversion‐fluorinated (M‐F), electrospinning‐fluorinated (E‐F), and attaching nanomaterials‐fluorinated (N‐F) membranes when feed water was a) salty feed, b) gypsum feed, c) organic brine, d) surfactant brine, e) surfactants organic brine, f) oily brine, g) oil‐in‐water emulsion, and h) real water and its mimics. Ini.: initial permeate flux, Fin.: final permeate flux, refers to the vapor flux after MD operation. Operation configuration: DCMD; feed temperature: mostly 60 °C; permeate temperature: mostly 20 °C; all operation durations were the flux‐stable time. Raw data are listed in Table S6 (Supporting Information).

NaCl (3.5 wt.% or 35 g L^−1^) typically refers to the average salinity observed in the Earth's oceans.^[^ ^83^ ^]^ This salinity level has been widely used to simulate seawater in MD salty feed. **Figure** **9**a and Table S6 (Supporting Information) showed that all membranes displayed satisfactory MD performance for the salty feed with stable permeate flux and conductivity/salt rejection. Indeed, pristine hydrophobic membranes (e.g., pristine PVDF membrane) can offer favorable performance, with the average and initial flux of 22.60 and 22.76 L m^−2^ h^−1^, respectively (Figure 9a). The average final conductivity of the permeate and final salt rejections were 4.38 µs cm^−1^ and >99.9%, respectively (Table S6, Supporting Information). Expectedly, the MD performance (mainly permeate flux) varied using the hydrophobic membrane (Figure 9a, Table S6, Supporting Information). In the DCMD equipped with an ENM, the permeate flux reached 57.5 L m^−2^ h^−1^ with a salt rejection of >99.80% during 100 h operation.^[^ ^43e^ ^]^ Due to the interconnected open structure, the pore size and porosity of the ENMs are much larger than those of membranes prepared by phase inversion, leading to the enhancement of MD behavior.^[^ ^46^, ^58^, ^84^ ^]^

However, some modified membranes showed a flux‐decline compared with the pristine one (Figure 9a). The average final flux of micromolding phase inversion and attaching nanomaterials membranes decreased to 19.03 and 21.73 L m^−2^ h^−1^, respectively (Figure 9a). Notably, the average permeate flux of the fluorinated membrane was the lowest (13.81 L m^−2^ h^−1^) (Figure 9a), indicating that COf might block up some membrane pores.

### 4.2 Gypsum Feed

Scaling‐induced wettability dramatically limits the application of MD for the desalination of high‐salinity feed, since the mineral crystals (typically gypsum) readily nucleate on the membrane surface due to temperature/concentration polarization.^[^ ^85^ ^]^ Unlike NaCl, lowly soluble CaSO_4_ could crystallize involving the hydrated reaction between Ca^2+^ and SO_4_^2−^ and then form the nasty gypsum scaling.^[^ ^85^, ^86^ ^]^ The obvious decrease in permeate flux can be observed. The final fluxes were relatively low (7.00–17.20 L m^−2^ h^−1^) compared to the initial average value (28.87 L m^−2^ h^−1^) (Figure 9b), and the permeate conductivity deteriorated from near‐zero to 306 µs cm^−1^ when gypsum feed was treated by DCMD equipped with pristine membranes.^[^ ^45k^ ^]^ CRlS membranes did not appear to be resistant to wetting caused by gypsum scaling without the assistance of fluorination (Figure 9b, Table S6, Supporting Information). After attaching nanomaterials‐fluorination, the average final flux and conductivity of the permeate were optimized to be 20.93 L m^−2^ h^−1^ and 8.32 µs cm^−1^, respectively (Figure 9b, Table S6, Supporting Information). Besides, Farid et al. ingeniously designed a simple approach for scaling inhibition in MD.^[^ ^87^ ^]^ The authors introduced nanobubbles with an average size of 128.81 nm into the feed channel, delaying the salt precipitation and crystal deposition on the pristine membrane surface.^[^ ^87^ ^]^ Overall, further investigation should focus on improving the ability of MD membranes to resist scaling‐induced wetting, involving the gypsum‐induced wetting that occurred when the bulk feed was supersaturated.^[^ ^85^, ^86^ ^]^

### 4.3 Organic and Surfactant Brine

Generally, algal organic matter in seawater comprises humic acid (HA), polysaccharides, and proteins, responsible for the wetting and fouling on the hydrophobic membrane surface during desalination by MD.^[^ ^59^ ^]^ To overcome the problem, attaching nanomaterials (‐fluorination) methods were widely used to enhance the anti‐wetting/fouling properties of the pristine MD membranes, resulting in an increase in the final permeate flux of 4–9 L m^−2^ h^−1^ (Figure 9c) and a decrease in the final permeate conductivity of 70–120 µs cm^−1^ (Table S6, Supporting Information).

Besides, surfactants with low surface tension are commonly used to test the anti‐wetting properties of the MD membrane. As shown in Figure 9d and Table S6 (Supporting Information), the introduction of surfactants in the salty feed reduced the permeate flux and salt rejection of the DCMD equipped with the pristine membrane, whose average values were 8.75 L m^−2^ h^−1^ and 43.61%, respectively (no available data for conductivity). Methods of electrospinning‐fluorination, attaching nanomaterials, and attaching nanomaterials‐fluorination can enhance the anti‐wetting properties of the hydrophobic membranes (Figure 9d, Table S6, Supporting Information). The average permeate flux of the corresponding membranes reached 17.49, 23.09, and 15.78 L m^−2^ h^−1^, respectively, close to their initial permeate fluxes (Figure 9d). In parallel high salt rejection could be achieved (Table S6, Supporting Information).

The surfactant organic brine was employed as the feed stream to mimic the seawater further. The pristine membrane could offer a final flux of 10.62 L m^−2^ h^−1^ (Figure 9e) and average permeate conductivity of 546.83 µs cm^−1^ (Table S6, Supporting Information). These values reached 35.05 L m^−2^ h^−1^ (Figure 9e), and 3.93 µs cm^−1^ (Table S6, Supporting Information), respectively, if the enhanced membranes modified via attaching nanomaterials‐fluorination were applied.

### 4.4 Oily Brine and Oil‐in‐Water Emulsion

For oily brine treatment, CRlS and CRlS‐COf effectively upgraded the MD performance. The final flux and salt rejection of DCMD equipped with pristine membranes were 7.18 L m^−2^ h^−1^ and 43%, respectively, far away from their initial values (Figure 9f, Table S6, Supporting Information). When the MD membrane was modified through attaching nanomaterials‐fluorination, the final flux of the permeate and salt rejection could achieve 16–27 L m^−2^ h^−1^ and 99.4%–100%, respectively (Figure 9f, Table S6, Supporting Information).

However, for the surfactant‐stabilized oil‐in‐water, the situation was quite different.^[^ ^45t^ ^]^ Without fluorination, CRlS can hardly prevent the MD membrane from wetting by oil‐in‐water emulsion (Figure 9g, Table S6, Supporting Information). For example, ENM would be rapidly wetted,^[^ ^43c^ ^]^ and exhibit poorer MD performance than the pristine membrane.^[^ ^46g^ ^]^

The most effective method for enhancing the MD membrane's anti‐emulsion‐wetting properties is to construct a Janus structure.^[^ ^45^, ^88^ ^]^ The Janus membrane comprises a hydrophobic/superhydrophobic bottom layer and a hydrophilic top coating. Hydrophilic coating is responsible for isolating oil and water to protect the bottom layer from wetting by the emulsion.^[^ ^45^, ^89^ ^]^ Moreover, the hydrophilic protective layer in water can effectively avoid gypsum scaling compared with the hydrophobic layer.^[^ ^90^ ^]^ Hence, the Janus membrane attracts increasing attention and is expected to become a promising MD membrane.

### 4.5 Real Water and its Mimics

Desalination involves removing salts, other minerals, and contaminants from seawater and wastewater to obtain fresh water for human consumption.^[^ ^91^ ^]^ For seawater desalinated by MD treatment, the CRlS (‐COf) membranes exhibited superior permeate flux and conductivity/salt rejection over the pristine one (Table S6, Supporting Information). For instance, Kharraz et al.^[^ ^41a^ ^]^ collected real seawater from the Hong Kong shoreline and desalinated it in a lab‐scale DCMD set‐up. The permeate flux of the pristine PVDF membrane sustained 19.5 L m^−2^ h^−1^ within the operation of 25 h and went down to 0 L m^−2^ h^−1^ after 97 h.^[^ ^41a^ ^]^ In contrast, CRlS (‐COf) membranes stabilized the permeate flux of 18.8–19 L m^−2^ h^−1^ and the salt rejection of 99.5%–99.7% at 133 h.^[^ ^41a^ ^]^ For desalinating imitative oil/gas production waste emulsion, the final flux and the permeate conductivity of the pristine membrane were 4.47 L m^−2^ h^−1^ and 601.8 µs cm^−1^.^[^ ^45t^ ^]^ These values were enhanced to 12.30 L m^−2^ h^−1^ and 210.72 µs cm^−1^, respectively, by using the attaching nanomaterials‐fluorinated membranes (Table S6, Supporting Information).

Li et al.^[^ ^45j^ ^]^ compared the MD performance of the membranes enhanced by attaching SiNPs with different grafting methods (i.e., electrostatic absorption and covalent bonding) and fluorination in the treatment of biologically pre‐treated coking wastewater. The permeate flux and conductivity of the two enhanced membranes were more stable than that of the pristine one in the DCMD process (Figure S2a, Supporting Information).^[^ ^45j^ ^]^ Zheng et al.^[^ ^45t^ ^]^ prepared an emulsion mixed with NaCl, SDS, and hexadecane in water, and desalinated it by DCMD equipped with the pristine, fluorinated, and attaching nanomaterials‐fluorinated membranes. As displayed in Figure S2b (Supporting Information), the permeate flux of the pristine membrane dropped rapidly to 0.25 L m^−2^ h^−1^. In contrast, the permeate conductivity increased exponentially to 601.8 µs cm^−1^, due to the wettability caused by SDS and hexadecane.^[^ ^45t^ ^]^ In contrast, the fluorinated membrane maintained stable flux and conductivity of the permeate within the DCMD operation of 700 min (Figure S2b, Supporting Information),^[^ ^45t^ ^]^ suggesting that surface chemical modification contributed to anti‐wetting properties of the membranes to a certain degree. The CRlS‐COf membrane (attaching nanomaterials) had stable permeate conductivity but low flux because the nanomaterials blocked the membrane pore.^[^ ^45t^ ^]^ A similar phenomenon was also reported by Li et al.^[^ ^45s^ ^]^

Consequently, methods to improve the anti‐wetting properties of hydrophobic membranes must be combined with the characteristics of the treated water. When feed matrices are highly soluble salts, pristine membranes are competent for the MD treatment. Otherwise, enhanced methods are required to prevent the membrane surface from wetting when feed solutions contain soluble gypsum, low‐surface‐tension surfactants and oil, oil‐in‐water emulsion, etc. Attaching nanomaterials‐fluorination is the most used method to improve the anti‐wetting properties of the hydrophobic membrane. The resultant membrane can handle the tricky feeds, however, at the expense of the permeate flux loss. Due to the open pore structure and reentrant‐like surface with low SFE of the resultant membrane, electrospinning‐fluorination might be a promising method and deserve further investigation.

## 5 Summary and Outlook

This review has outlined recent advances in some methods, named CRlS, COf, and CRlS‐COf, which significantly enhance the anti‐wetting properties of the hydrophobic membranes and offer the possibility to improve the performance of the MD process. The conclusions are as follows:

1) With the application of MD in water treatment, the research on membrane wetting is increasing year by year (Figure 1). Some parameters, such as WCA, LEP, and SFE, have been widely used to characterize membrane wetting. Among them, WCA directly reflects the hydrophobic properties of the membrane surface. LEP and SFE, as supplemental parameters, represent the anti‐wetting properties of MD membranes together with WCA. Some parameters, such as the dynamic CA characterizing the adhesion interaction between droplet and membrane, need further study.
2) Commercial hydrophobic membranes with high WCA of >90° are generally made from polymer plastic, such as PVDF, PTFE, PVDF‐HFP, PP, PES, and PE (Figure 2). Inspired by the reentrant structure, many CRlS methods (i.e., surface structural construction) to improve the membrane hydrophobicity emerged, which could be classified as micromolding phase inversion, electrospinning, and attaching nanomaterials. CRlS efficiently improved the WCA of the hydrophobic membranes. Surface chemical modification (usually COf) helped to enhance the anti‐wetting properties of hydrophobic membranes. Typically, COf decreased the SFE of the membrane, thereby increasing the WCA. After CRlS and/or COf, the WCA of most hydrophobic membranes exceeded 120° and even reached 150° (e.g., attaching nanomaterials‐fluorinated membranes) (Figures 4 and 7).
3) Generally, enhanced MD membranes showed more excellent salt rejection than pristine ones. CRlS (except for electrospinning) and/or COf may lower the permeate fluxes, but improve the MD membrane's hydrophobicity and enhance the anti‐wetting properties against many feeds, except for the oil‐in‐water emulsion. In treating salty feed, the enhanced MD membranes had no obvious advantage over the pristine ones, except for the large flux of ENM. When the feed matrices were salty feed mixed with low surface tension components, the superiority of enhanced MD membrane was obvious (Figure 9, Table S6, Supporting Information).

It is worth noting that most enhanced anti‐wetting methods of the hydrophobic membrane are still in the laboratory, with some issues to be worked out. For example, the scaling‐up of membranes attaching nanomaterials (e.g., SiNPs) probably suffers discrete distribution and potential exfoliation of nanoparticles.^[^ ^45k^ ^]^ Besides, research should be encouraged to invent more robust MD membranes. The term “robust” stands for the superhydrophobic surface that can accommodate a variety of feed streams and maintain considerable flux and salt rejection. The facile strategies for constructing bionic membranes should be further developed, to achieve ranked‐reentrant‐structure array like springtail cuticle morphology instead of disorderly structure accumulation.

## Conflict of Interest

The authors declare no conflict of interest.

## Supporting information

Supporting Information
