# Supplementary material for: Enhanced Anti‐Wetting Methods of Hydrophobic Membrane for Membrane Distillation
Source: Adv Sci (Weinh). 2023 May 23;10(23):2300598. doi: 10.1002/advs.202300598 (PMC10427381; doi:10.1002/advs.202300598)
Supplement: Supplementary file 1 — Supporting Information [file ADVS-10-2300598-s001.pdf]

ADVANCED SCIENCE
Open Access

Supporting Information

for *Adv. Sci.*, DOI 10.1002/advs.202300598

Enhanced Anti-Wetting Methods of Hydrophobic Membrane for Membrane Distillation

*Honglong Zhang and Xuan Zhao**

Supporting Information

Enhanced Anti-Wetting Methods of Hydrophobic Membrane for Membrane Distillation

Honglong Zhang, Xuan Zhao*

H. L. Zhang, X. Zhao

Lab of Environmental Science & Technology, INET, Tsinghua University, Beijing 100084, People's Republic of China

Email: zhxinet@tsinghua.edu.cn

Totally 70 pages, including 6 Tables and 2 Figures

Table S1. Wetting parameters of various MD membranes.

Membrane types	Enhanced methods	WCA (°)	LEP (kPa)	Roughness[a)] (nm)		SFE calculation		SFE (mN m^{-1})	Ref.
				Ra	Rq	Methods	Probe liquids		
PVDF FSM[b)]	None	115.46 ± 1.66	110.0 ± 1.5	550	/	OWRK	Water and diiodomethane	29.02 ± 0.83	[1]
PVDF FSM	None	114 ± 2	229	/	188.3	/	/	30.3	[2]
PVDF FSM	None	130.7	40.4	/	/	/	/	/	[3]
PVDF FSM	None	128	376.0	/	/	/	/	25	[4]
PVDF FSM	None	119.0	/	570	/	OWRK	Water and diiodomethane	29.01	[5]
PVDF FSM	None	/	/	/	/	/	/	30.3	[6]
PVDF FSM	None	132.5	/	/	/	/	/	/	[7]
PVDF FSM	None	/	200	/	/	/	/	/	[8]
PVDF FSM	None	127	/	/	/	/	/	/	[9]
PVDF FSM	None	126	238 ± 5	/	/	/	/	/	[10]
PVDF FSM	None	120 ± 1	/	/	50 ± 2	OWRK	Water and glycerinum	41.6 ± 0.3	[11]
PVDF FSM	None	139.2 ± 3.7	/	/	/	OWRK	Water and diiodomethane	71.8 ± 2.4	[12]
PVDF FSM	None	88.2	/	155	196	LW-AB	Water, formamide, and diiodomethane	38.67	[13]
PVDF FSM	None	125.7 ± 2.8	/	195	316	LW-AB	Water, glycerinum, and diiodomethane	14.11	[14]
PVDF FSM	None	125.5	535 ± 28	190 ± 8	253 ± 13	LW-AB	Water, glycerinum, and diiodomethane	9.50	[15]
PVDF FSM	None	118	/	460	/	/	/	/	[16]

PVDF FSM	None	125	249	/	100.78	/	/	/	[17]
PVDF FSM	None	130.1 ± 1.2	/	/	/	/	/	/	[18]
PVDF FSM	None	97.7	16.61	/	104	OWRK	/	21.23	[19]
PVDF FSM	None	130.1 ± 1.2	237 ± 3	/	/	OWRK	Water and diiodomethane	65.8 ± 3.2	[20]
PVDF FSM	None	109	72.37	/	122	/	/	69.58	[21]
PVDF FSM	None	115.46 ± 1.17	130 ± 2	610	/	OWRK	Water and diiodomethane	29.02 ± 0.27	[22]
PVDF FSM	None	125 ± 1	120	/	/	LW-AB	Water, glycerinum, and diiodomethane	14 ± 0.4	[23]
PVDF FSM	None	114.7 ± 4.4	208	/	/	/	/	/	[24]
PVDF FSM	None	126.7 ± 1.4	240	98.6	125	/	/	/	[25]
PVDF FSM	None	83.5	140	/	/	Equation of state	/	27.5	[26]
PVDF FSM	None	123.1	188	780	/	/	/	/	[27]
PVDF FSM	None	110.2	74.40	/	111 ± 6	OWRK	/	54.3	[28]
PVDF FSM	None	115	/	/	/	/	/	/	[29]
PVDF FSM	None	127.0 ± 1.0	250	148.0 ± 6.0	189.0 ± 5.0	/	/	/	[30]
PVDF FSM	None	125.0	240	101.0 ± 3.0	132.0 ± 2.0	/	/	/	[31]
PVDF FSM	None	126 ± 1.6	/	80.83	/	/	/	/	[32]
PVDF FSM	None	120.17 ± 2.1	/	/	/	LW-AB	Water, ethylene glycol, and diiodomethane	21.71	[33]

PVDF FSM	None	136.3 ± 1.0	315 ± 18	132	176	LW-AB	Water, glycerinum, and diiodomethane	14.31	[34]
PVDF FSM	None	101.2 ± 10.9	/	/	233.8 ± 18.6	OWRK	Water, glycerinum, and diiodomethane	22 ± 0.15	[35]
PVDF FSM	None	132.2 ± 1.6	/	123.0 ± 10.3	/	/	/	/	[36]
PVDF FSM	None	110	98.61	890	1030	OWRK	/	54.15	[37]
PVDF FSM	None	98.47 ± 5.1	/	21.13	27.38	LW-AB	Water, glycerinum, and diiodomethane	29.51	[38]
PVDF FSM	None	128.4 ± 1.7	105.2 ± 2.5	/	/	/	/	/	[39]
PVDF FSM	None	119.4	146.6	85	/	OWRK	/	29.52 ± 0.88	[40]
PVDF FSM	None	121.48	/	/	136	/	/	/	[41]
PVDF FSM	None	114 ± 2	/	/	49.68	OWRK	Water and glycerinum	46.3 ± 1.7	[42]
PVDF FSM	None	121.29	242.58	/	/	/	/	/	[43]
PVDF FSM	None	127.0 ± 1.0	250	/	/	/	/	/	[44]
PVDF FSM	None	110	/	/	/	/	/	/	[45]
PVDF FSM	None	118.7	/	/	/	/	/	/	[46]
PVDF FSM	None	124.2	160	/	112	OWRK	Water and diiodomethane	13.68	[47]
PVDF FSM	None	124.2 ± 1.2	/	/	/	LW-AB	Water, glycerinum, and diiodomethane	13.68	[48]
PVDF FSM	None	98.5 ± 2.1	/	/	/	/	/	/	[49]
PVDF FSM	None	132 ± 2	93 ± 4	/	/	OWRK	Water, glycerinum, 1-bromonaphthalene,	8.67	[50]

							and n-hexadecane		
PVDF FSM	None	123	114 ± 2	/	/	/	/	/	[51]
PVDF FSM	None	120.3 ± 1.2	/	597.6	/	/	/	30.28	[52]
PVDF FSM	None	124	204	/	/	OWRK	Water and diiodomethane	11.5	[53]
PVDF FSM	None	119.8 ± 1.5	190	/	/	/	/	/	[54]
PVDF FSM	None	114.6 ± 1.2	88.91	70 ± 4	/	OWRK	Water and n-hexane	54.37	[55]
PVDF FSM	None	117 ± 3	205 ± 8.66	/	/	OWRK	Water and diiodomethane	11.55 ± 3.80	[56]
PVDF FSM	None	135 ± 1.5	223 ± 5	126	164	/	/	/	[57]
PVDF FSM	None	113.75 ± 1.21	/	/	/	/	/		[58]
PVDF FSM	None	83<br>83<br>77.48	735<br>622<br>700	/	/	LW-AB	Water, ethylene glycol, and diiodomethane	37.30<br>39.33<br>40.09	[59]
PVDF FSM	None	120<br>122<br>149	230<br>110<br>200	/	/	/	/	/	[60]
PVDF FSM	None	/	204<br>229<br>105<br>110	/	/	/	/	/	[61]
PVDF FSM	None	83.65<br>78.54<br>77.14	729.4<br>671.7<br>424.45	/	/	LW-AB	Water, ethylene glycol, and diiodomethane	37.29<br>39.56<br>42.35	[62]

		74.91	473.9					35.31	
		76.88	585.17					42.06	
		88.35	663.46					34.37	
		70.62	754.12					39.82	
		80.21	782.97					37.08	
		77.48	700.55					40.06	
PVDF FSM	Fluorination	110.3	/	/	/	LW-AB	Water, ethylene glycol, and diiodomethane	11.9	[63]
PVDF FSM	Fluorination	135.79 ± 1.32	/	/	/	/	/	/	[58]
PVDF FSM	Fluorination	139	146 ± 2	/	/	/	/	/	[51]
		149	175 ± 5						
PVDF FSM	Attaching SiNPs-fluorination	169.0 ± 1.5	550	/	/	/	/	/	[44]
PVDF FSM	Attaching SiNPs-fluorination	159.79	/	/	/	/	/	/	[45]
PVDF FSM	Attaching SiNPs-fluorination	153.5	/	/	/	/	/	/	[46]
PVDF FSM	Attaching fluorinated SiNPs	167.3	250	/	138	OWRK	Water and diiodomethane	0.79	[47]
PVDF FSM	Attaching fluorinated SiNPs	167.3 ± 0.6	/	/	/	LW-AB	Water, glycerinum, and diiodomethane	0.79	[48]
PVDF FSM	Attaching fluorinated SiNPs	170.8 ± 1.3	/	/	/	/	/	/	[49]
PVDF FSM	Attaching SiNPs-fluorination	152.0 ± 1.4	200	/	/	/	/	/	[54]
		166.5 ± 1.4	210						
PVDF FSM	Attaching SiNPs-fluorination	162.92	/	/	213	/	/	/	[41]
		161.50			174				

PVDF FSM	Attaching SiNPs-fluorination	135	/	/	/	/	/	/	[29]
		138							
		162							
PVDF FSM	Attaching SiNPs-fluorination	155.4 ± 1.4	>190	/	/	/	/	/	[64]
		158.0 ± 3.1	>170						
		155.2 ± 3.3	/						
PVDF FSM	Attaching SiNPs-fluorination	138.07	269.52	/	/	/	/	/	[43]
		162.58	326.26						
		155.48	309.02						
		154.84	301.03						
PVDF FSM	Attaching fluorinated SiNPs	139.7	/	1710	/	OWRK	Water and diiodomethane	2.16	[5]
		141.5		1910				1.93	
		151.2		2170				0.82	
		138.0		1630				2.29	
		138.2		2000				1.89	
		131.6		1400				2.93	
		134.1		1690				1.99	
PVDF FSM	Fluorination	147.8 ± 3.8	265	92.1	116	/	/	/	[25]
	Attaching SiNPs-fluorination	155.7 ± 2.6	285	142	192				
PVDF FSM	Fluorination	144.50	337	/	107.30	/	/	/	[17]
	Attaching fluorinated SiNPs	158.44	372		138.91				
		135.70	317		156.70				
		164.40	409		181.93				
PVDF FSM	Attaching methylate SiNPs	93.7	/	120.488	168.401	LW-AB	Water, glycerinum, and diiodomethane	38.95	[65]
	Incorporating PTFE-attaching	115.5	/	232.239	319.76			38.95	

	methylate SiNPs								
PVDF FSM	Attaching ZnO nanorods-fluorination	152	277 ± 8	/	/	/	/	/	[10]
PVDF FSM	Fluorination	134.21 ± 2.52	139 ± 7	880	/	OWRK	Water and diiodomethane	4.86 ± 0.15	[22]
	Fluorination-Attaching fluorinated ZnO nanoparticles	153.03 ± 3.10	189 ± 4	1370				0.78 ± 0.14	
PVDF FSM	Attaching TiO_2 nanoparticles-fluorination	168.0 ± 2.0	420	203.0 ± 5.0	255.0 ± 3.0	/	/	/	[30]
PVDF FSM	Fluorination	146 ± 5	130	/	/	LW-AB	Water, glycerinum, and diiodomethane	0.5 ± 0.14	[23]
	Attaching TiO_2 nanoparticles-fluorination	163 ± 3	190					0.1 ± 0.01	
PVDF FSM	Incorporating TiO_2 nanoparticles-fluorination	162.4 ± 0.8	/	/	/	/	/	/	[66]
		165.2 ± 1.0							
		163.6 ± 1.5							
		162.9 ± 0.8							
		151.2 ± 0.5							
		158.7 ± 1.5							
		163.2 ± 1.6							
		162.7 ± 0.4							
		163.2 ± 1.1							
		165.3 ± 1.0							
PVDF FSM	Coating polyethylene glycol-He plasma treatment	40.9 ± 0.8	/	/	/	LW-AB	Water, ethylene glycol, and diiodomethane	47.36	[33]
	Coating polyethylene glycol-He	25.2 ± 0.7						54.23	

	plasma treatment-attaching TiO_2 particles-He plasma treatment								
PVDF FSM	Attaching Ag nanoparticles-fluorination	155.2 ± 1.6	/	314.78	/	/	/	/	[32]
PVDF FSM	Fluorination	136.4 ± 3.3	223	/	/	/	/	/	[24]
	Attaching FeOOH nanoparticles-fluorination	149.1 ± 4.4	292						
PVDF FSM	Incorporating Fe_3O_4 nanoparticles	99.2	22.69	/	79	OWRK	/	25.33	[19]
	Incorporating Fe_3O_4 nanoparticles-HCl leaching	100.1	/		88			37.73	
PVDF FSM	Attaching SiO_2 microparticles	159.5 ± 1.1	353 ± 15	402	516	LW-AB	Water, glycerinum, and diiodomethane	8.91	[34]
PVDF FSM	Attaching carbon black nanoparticles	55.1 ± 7.0	/	/	228 ± 63.7	OWRK	Water, glycerinum, and diiodomethane	42 ± 0.25	[35]
PVDF FSM	Attaching carbon black nanoparticles-fluorination	140.8 ± 2.1	/	109.2 ± 19.0	/	/	/	/	[36]
PVDF FSM	Attaching single-walled carbon nanohorns	165.3	196.04	1010	1250	OWRK	/	15.99	[37]
PVDF FSM	Fluorination	155	172.40	/	291 ± 11	OWRK	/	7.31	[28]
	Silanization-attaching multi-walled carbon nanotubes	140	141.06		615 ± 22			27.20	
PVDF FSM	Incorporating chlorotrifluoroethylene (CTFE)	105.37 ± 3.4	/	32.39	41.84	LW-AB	Water, glycerinum, and diiodomethane	25.68	[38]
PVDF FSM	Incorporating fluorinated melamine	115.4	200	/	/	Equation of state	/	10.0	[26]

PVDF FSM	Micromolding phase inversion	130	229	2450	/	/	/	/	[27]
	Micromolding phase inversion-fluorination	154.5	321	/					
PVDF FSM	Micromolding phase inversion	166.0 ± 2.3	/	/	/	OWRK	Water and diiodomethane	47.3 ± 0.6	[12]
	Micromolding phase inversion-fluorination	175.6 ± 1.3						0.27 ± 0.12	
PVDF FSM	Micromolding phase inversion	155.3 ± 1.7	/	/	/	/	/	/	[18]
	Micromolding phase inversion-fluorination	166.8 ± 1.5							
PVDF FSM	Incorporating CTFE-micromolding phase inversion	130	/	/	/	/	/	/	[67]
	Incorporating CTFE-micromolding phase inversion-fluorination	140							
		150							
PVDF FSM	Micromolding phase inversion	155.3 ± 1.7	239 ± 2	/	/	OWRK	Water and diiodomethane	43.9 ± 1.4	[20]
	Micromolding phase inversion-attaching SiNPs- fluorination	175.6 ± 2.1	307 ± 12					0.24 ± 0.1	
PVDF FSM	Silanization	120	164.4	209	/	OWRK	/	28.43 ± 0.50	[40]
	Silanization-grafting chitosan	164.8	320.9	260				20.51 ± 0.65	
PVDF FSM	Silanization	131	133.07	/	250	/	/	32.48	[21]
	Silanization-attaching multi-walled carbon nanotubes	152	218.50		705			10.32	
PVDF FSM	Silanization	130 ± 1.2	236.33	346 ±	/	OWRK	Water and n-hexane	22.26	[55]

				21					
	Silanization-grafting chitosan	157.4 ± 1.4	436.66	386 ± 23	386 ± 26			22.52	
	Grafting silanized chitosan	168.2 ± 1.6	629.30	440 ± 26	440 ± 32			6.59	
PVDF FSM	Attaching TiO_2-coated carbon nanofibers	/	300	155.0 ± 2.5	196.0 ± 3.0	/	/	/	[31]
		/	320	207.0 ± 3.5	280.0 ± 2.0				
		172.0	350	346.0 ± 3.0	451.0 ± 3.0				
		/	391	389.0 ± 2.5	469.0 ± 5.0				
		/	400	362.0 ± 8.0	448.0 ± 6.0				
PVDF FSM	Attaching polydimethylsiloxane (PDMS)/PVDF microbeads	156.7 ± 1.9	125.2 ± 2.1	/	/	/	/	/	[39]
		156.8 ± 2.5	126.3 ± 1.6						
		157.1 ± 1.7	128.4 ± 2.3						
		159.8 ± 3.5	128.5 ± 1.8						
		162.1 ± 3.2	129.1 ± 0.9						
PVDF FSM	Attaching PDMS-polyhedral oligomeric silsesquioxane (POSS)/PVDF cauliflower-shaped beads	150	/	1940	/	/	/	/	[16]
		155		2390					
		166		3480					
		157		3510					
		152		2720					

PVDF HFM[c)]	None	117.14	/	173 ± 39	133 ± 32	Fowkes	Water and ethylene glycol	18.8	[68]
PVDF HFM	None	/	270	/	/	/	/	35.7	[69]
PVDF HFM	None	92.6	172	/	/	/	/	/	[70]
PVDF HFM	Attaching SiNPs-fluorination	145.34	/	175 ± 19	133 ± 16	Fowkes	Water and ethylene glycol	14.0	[68]
PVDF ENM[d)]	Electrospinning	142.61	45.1 ± 0.3	/	/	/	/	/	[71]
		148.77	42.8 ± 0.2						
		140.45	43.7 ± 0.3						
		145.69	45.3 ± 0.4						
PVDF ENM	Electrospinning	148.4 ± 2.4	/	/	/	/	/	32.49 ± 0.38	[72]
		148.0 ± 1.7	51.0 ± 2.8					32.42 ± 0.25	
		146.3 ± 2.6	62.5 ± 2.1					32.12 ± 0.36	
		145.7 ± 2.7	74.0 ± 1.4					32.02 ± 0.38	
		145.3 ± 0.6	46.5 ± 0.7					32.41 ± 1.12	
		143.9 ± 4.0	52.0 ± 1.4					32.27 ± 0.74	
		143.1 ± 3.4	65.5 ± 2.1					32.50 ±	

								0.78	
PVDF ENM	Electrospinning	145.3 ± 0.6	46.5 ± 0.7	/	/	/	/	/	[73]
		145.8 ± 0.5	52.0 ± 1.4						
		146.4 ± 0.7	55.5 ± 2.1						
		149.0 ± 1.2	54.5 ± 2.1						
		147.0 ± 1.4	51.5 ± 2.1						
		145.3 ± 0.6	46.5 ± 0.7						
		145.3 ± 0.6	46.5 ± 0.7						
		146.3 ± 0.8	50.0 ± 1.4						
		147.6 ± 1.4	53.0 ± 1.4						
		145.8 ± 1.2	48.5 ± 2.1						
		147.1 ± 1.0	55.5 ± 0.7						
		148.1 ± 0.6	60.0 ± 1.4						
		149.5	64.5 ± 0.7						
		146.6 ± 0.5	66.5 ± 0.7						
		148.9 ± 0.8	68.5 ± 2.1						
		149.6 ± 0.9	70.5 ± 0.7						
		151.7 ± 1.4	73.0 ± 1.4						
PVDF ENM	Electrospinning	132 ± 1	54	/	/	/	/	/	[74]
PVDF ENM	Electrospinning-attaching SiNPs-fluorination	169 ± 1	210	/	/			/	
PVDF ENM	Electrospinning	129 ± 2	86 ± 3	/	/	/	/	/	[75]
PVDF	Electrospinning-attaching	163 ± 3	147 ± 12	/	/			/	

ENM	silanized SiNPs								
PVDF ENM	Electrospinning-fluorination	132.2 ± 1.9	67 ± 3	/	194.2	/	/	/	[76]
PVDF ENM	Incorporating SiNPs-electrospinning-fluorination	154.6 ± 2.2	143 ± 4	/	230.4			/	
PVDF ENM	Electrospinning	138.0 ± 2.3	84	840	/	LW-AB	Water, glycerinum, and diiodomethane	35.19	[77]
PVDF ENM	Electrospinning-attaching ZnO nanorods- fluorination	161.3 ± 2.8	220	2270	/			/	
PVDF ENM	Electrospinning	131.7	83	2220	2890	/	/	/	[78]
PVDF ENM	Electrospinning-attaching ZnO nanorods- fluorination	164.9	225	4190	5420			/	
PVDF ENM	Electrospinning	142.7 ± 2.9	80	/	/	/	/	/	[79]
PVDF ENM	Electrospinning-attaching Ag nanoparticles- fluorination	165 ± 1	220	/	/			/	
PVDF ENM	Electrospinning	135	/	/	/	OWRK	Water and hexadecane	48	[80]
PVDF ENM	Electrospinning-coating polyaniline-fluorination	155	/	/	/			21	
PVDF ENM	Electrospinning	132.65 ± 0.71	38	4.87	3.01	/	/	/	[81]
PVDF ENM	Electrospinning-fluorination	148.41 ± 1.50	70	1.76	1.21			/	
PVDF	Electrospinning	136.1 ± 1.2	240	/	/	/	/	/	[82]

ENM									
PVDF ENM	Electrospinning-fluorination	160.2 ± 2.0	245	/	/			/	
PVDF ENM	Electrospinning	133.6 ± 1.8	142.7±1.2	/	/	LW-AB	Water, methanol, ethylene glycol, and diiodomethane	25	[83]
PVDF ENM	Electrospinning-fluorination	160.6 ± 4.5	186.7 ± 1.6	/	/			0.503	
PVDF ENM	Electrospinning	145.37	164.4	9.35	/	/	/	/	[84]
PVDF ENM	Electrospinning-silanization	151.08	197.6	11.9	/			/	
PVDF ENM	Electrospinning-attaching silanized SiNPs	159.90	257.1	46.3	/			/	
PVDF ENM	Electrospinning	94 ± 3	/	104	136	LW-AB	Water, glycerinum, and diiodomethane	8.41	[85]
PVDF ENM	Incorporating SiNPs-electrospinning	113 ± 5	/	/	/			11.2	
PVDF ENM	Incorporating silanized SiNPs-electrospinning	156 ± 6	/	141	172			9.86	
PVDF ENM	Incorporating tetrabutylammonium hexafluorophosphate-electrospinning	138.2	180	705	/	/	/	/	[86]
PVDF ENM	Incorporating polystyrene-electrospinning	137.8	40	661	/			/	
PVDF	Incorporating	151.7	120	1607	/			/	

ENM	tetrabutylammonium hexafluorophosphate and polystyrene-electrospinning								
PVDF ENM	Electrospinning	131.8	23.9	/	/	/	/	/	[87]
PVDF ENM	Co-electrospinning to form beaded nanofiber	138	53.8	/	/	/	/	/	
		141	65.2						
		142	76.1						
		144	142.7						
		148	107.6						
		136.2 ± 0.22	47.5						
		139.6 ± 0.16	58.4						
		141.1 ± 0.26	58.1						
		141.4 ± 0.12	51.7						
		141.9 ± 0.68	64.0						
PTFE FSM	None	140	360	171	219	/	/	/	[88]
PTFE FSM	None	118	461.95	/	/	OWRK	Water, ethylene glycol, glycerinum, and propanol	5.54	[89]
PTFE FSM	None	95.6	/	165	212	LW-AB	Water, formamide, and diiodomethane	41.62	[13]
PTFE FSM	None	141.6 ± 1.5	/	177	209	LW-AB	Water, glycerinum, and diiodomethane	9.22	[14]
PTFE FSM	None	125 ± 2	282	/	190.5	/	/	9.1	[2]
PTFE FSM	None	135.3	544 ± 34	208 ± 3	257 ± 11	LW-AB	Water, glycerinum,	10.22	[15]

							and diiodomethane		
PTFE FSM	None	118	/	/	/	/	/	/	[90]
PTFE FSM	None	151.7 ± 6.5	338	/	/	/	/	/	[91]
PTFE FSM	None	119 ± 1	/	/	/	/	/	/	[92]
PTFE FSM	None	118	/	/	/	/	/	/	[93]
PTFE FSM	None	114	/	/	/	/	/	/	[94]
PTFE FSM	None	/	207	/	/	/	/	/	[95]
PTFE FSM	None	124.2	/	/	/	/	/	/	[96]
PTFE FSM	None	125 ± 2	282	/	/	/	/	9.1	[2]
PTFE FSM	None	132.5	/	128	154	/	/	/	[97]
PTFE FSM	None	122 ± 5	654.209	/	/	/	/	/	[98]
PTFE FSM	None	134.1	33.1	/	/	/	/	/	[3]
PTFE FSM	None	142	478.0	/	/	/	/	19	[4]
PTFE FSM	None	137	>400	/	/	/	/	/	[99]
PTFE FSM	None	/	/	/	/	/	/	19.1	[6]
PTFE FSM	None	105	/	/	248	/	/	/	[100]
PTFE FSM	None	136	900	/	/	/	/	/	[101]
PTFE FSM	None	142	/	/	/	/	/	/	[9]
PTFE FSM	None	145<br>133.2	335<br>350	123.1<br>68.2	/	LW-AB	Water, glycerinum, and diiodomethane	9.39<br>11.23	[102]
PTFE FSM	None	119 ± 2<br>138 ± 2	/	/	63.81<br>87.91	OWRK	Water and glycerinum	37.4 ± 1.3<br>20.3 ± 0.9	[42]
PTFE FSM	None	121 ± 1<br>133 ± 1	/	/	70 ± 2<br>65 ± 2	OWRK	Water and glycerinum	42.3 ± 0.3<br>38.0 ± 0.3	[11]
PTFE FSM	None	121 ± 1	/	/	70 ± 2	OWRK	Water, glycerinum,	42.3 ± 0.3	[103]

		133 ± 1			65 ± 2		and diiodomethane	29.9 ± 0.3	
PTFE FSM	None	140 ± 3	103.42	/	/	/	/	/	[104]
		160 ± 3	75.84						
PTFE FSM	None	136.1	360	/	/	/	/	/	[105]
		119.0	350						
		145.9	320						
PTFE FSM	None	140	/	/	/	/	/	/	[106]
		148							
		150							
PTFE FSM	None	141.9	/	/	/	/	/	/	[107]
		133.9							
		126.6							
		135.5							
PTFE FSM	None	135	260	/	/	/	/	/	[60]
		138	350						
		140	400						
		133	370						
		139	350						
		134	360						
PTFE FSM	None	/	282	/	/	/	/	/	[61]
			276						
			138						
			368						
			288						
			463						

			280						
			124						
PTFE FSM	None	115.6	117.72	/	/	/	/	/	[108]
		120.1	75.67						
		114.7	37.42						
		124.4	82.66						
		124.8	83.33						
		125.2	48.8						
		132.2	152.5						
		133.5	96.44						
		133.6	54.45						
PTFE FSM	Incorporating PVDF	138.6	516 ± 25	231 ± 6	306 ± 17	LW-AB	Water, glycerinum, and diiodomethane	9.83	[15]
PTFE FSM	Attaching carbon nanotube	112	441.26	/	/	OWRK	Water, ethylene glycol, glycerinum, and propanol	7.64	[89]
	Attaching graphene oxide	108	434.37					8.77	
PTFE ENM	Incorporating poly(ethylene oxide)-electrospinning	144 ± 2	115 ± 2	/	/	/	/	/	[109]
PTFE ENM	Incorporating PAN-electrospinning-attaching SiNPs-fluorination	166.9	220	3467	/	/	/	/	[110]
PVDF-HFP FSM	None	/	294	/	/	/	/	/	[111]
PVDF-HFP FSM	None	90.1	/	/	/	/	/	/	[112]

PVDF-HFP FSM	None	110	296.47	174	209	/	/	/	[113]
PVDF-HFP FSM	None	100.4	/	/	/	/	/	/	[114]
PVDF-HFP FSM	None	118	/	348	/	/	/	/	[115]
PVDF-HFP FSM	None	113.2	/	161.3	/	/	/	/	[116]
PVDF-HFP FSM	None	109	/	292	388	/	/	/	[117]
PVDF-HFP FSM	None	126	/	19	/	/	/	/	[118]
PVDF-HFP FSM	None	90.1 ± 1.7	/	/	/	/	/	/	[119]
PVDF-HFP FSM	None	134.6	/	1680	/	/	/	8.19	[120]
PVDF-HFP FSM	None	115.2 ± 2.0	280	/	/	/	/	/	[121]
PVDF-HFP FSM	None	98.6	/	1.27	/	/	/	/	[122]
PVDF-HFP FSM	None	124 ± 2	270	/	/	Geometric mean	/	5.46	[123]
PVDF-HFP FSM	None	134.43 ± 1.38	484	/	/	Geometric mean	/	8.17 ± 0.29	[124]
PVDF-HFP FSM	None	134 ± 1.08	/	1680	/	OWRK	Water and diiodomethane	8.17 ± 0.29	[35b, 120]

PVDF-HFP FSM	Attaching fluorinated SiNPs	174 ± 3	550	/	/		/	0.3	[123]
PVDF-HFP FSM	Attaching silanized SiNPs	161 ± 1.98	/	3860	/	OWRK	Water and diiodomethane	0.68 ± 0.09	[35b, 120]
PVDF-HFP FSM	Attaching silanized SiNPs	136.87	459	/	/	Geometric mean	/	7.28 ± 0.22	[124]
		138.75	423					6.42 ± 0.20	
		139.67	478					5.47 ± 0.29	
		143.46	520					5.34 ± 0.13	
		145.98	558					4.78 ± 0.13	
		149.87	595					4.68 ± 0.10	
PVDF-HFP HFM	None	115 ± 1.5	112 ± 4.5	44	/	/	/	/	[125]
PVDF-HFP ENM	Electrospinning	135.9 ± 4.7	43 ± 2.5	/	/	/	/	/	[126]
PVDF-HFP ENM	Incorporating fluorinated POSS-electrospinning	148	102.0	1240	/	/	/	/	[127]
PVDF-HFP ENM	Electrospinning-attaching PDMS/PVDF microbeads	167.91 ± 4.00	129.5 ± 3.4	5040	/	OWRK	Water and diiodomethane	4.18 ± 0.27	[1]
PVDF-HFP ENM	Electrospinning	110.07	/	/	/	/	/	/	[128]
PVDF-HFP ENM	Electrospinning-*co*-electrospray SiNPs/PVDF-HFP	162	/	/	/			/	
PVDF-HFP ENM	Electrospinning	143.8 ± 2.5	145	284.65 ± 28.71	358.71 ± 37.64	/	/	/	[129]
PVDF-HFP	Co-axial electrospinning PVDF-	169.7 ± 0.7	197	488.43	633.97 ±			/	

ENM	HFP and SiO_2			± 221.39	282.05				
PVDF-HFP ENM	Electrospinning	126	/	/	/	/	/	/	[130]
PVDF-HFP ENM	Incorporating fluorinated SiNPs-electrospinning	148	/	/	/			/	
PVDF-HFP ENM	Electrospinning	130.6	119	894	/	/	/	/	[131]
PVDF-HFP ENM	Incorporating (3-aminopropyl)triethoxysilane (APTES)-electrospinning-attaching SiNPs-fluorination	151.49	223	1737	/			/	
PVDF-HFP ENM	Electrospinning	130	/	/	/	/	/	/	[132]
PVDF-HFP ENM	Incorporating benzyltriethylammonium chloride-electrospinning-attaching SiNPs-fluorination	150	/	/	/			/	
PVDF-HFP ENM	Incorporating fluoroalkylsilanes-electrospinning	135.3 ± 1.2	101	2310	/	OWRK	Water and diiodomethane	26.85 ± 0.63	[133]
PVDF-HFP ENM	Electrospinning-attaching fluorinated ZnO nanoparticles	161.4 ± 3.4	187	3260	/			0.75 ± 0.43	
PVDF-HFP ENM	Electrospinning	121.3 ± 1.5	117 ± 3	/	/	OWRK	Water and ethylene glycol	38.3 ± 1.4	[134]
PVDF-HFP ENM	Electrospinning-attaching TiO_2 nanorods-fluorination	152.6 ± 1.2	254 ± 12	/	/			11.2 ± 1.2	

PVDF-HFP ENM	Electrospinning	140 ± 1.9	/	2880	/	/	/	25.61	[52]
PVDF-HFP ENM	Electrospinning-attaching fluorinated TiO_2 nanoparticles	157 ± 1.6	/	4930	/			11.42	
PVDF-HFP ENM	Electrospinning	139.2 ± 0.11	121	2410	/	/	/	22.4 ± 0.07	[135]
PVDF-HFP ENM	Electrospinning-*co*-electrospray PVDF-HFP nanobeads	153.8 ± 0.19	217	8620	/			12.6 ± 0.09	
PVDF-HFP ENM	Electrospinning	140 ± 2	84 ± 4	/	/	OWRK	Water, glycerinum, 1-bromonaphthalene, and n-hexadecane	9.14	[50]
PVDF-HFP ENM	Incorporating PDMS-electrospinning	143 ± 2	103 ± 4	/	/			9.71	
PVDF-HFP ENM	Electrospinning	144.9 ± 0.3	165	115	/	OWRK	Water and diiodomethane	0.990	[136]
PVDF-HFP ENM	Electrospinning-fluorination	154.1 ± 0.1	225	159	/			0.372	
PVDF-HFP ENM	Electrospinning	146.20	/	/	405	Fowkes	Water and diiodomethane	2.19	[137]
PVDF-HFP ENM	Co-electrospinning polyethylene terephthalate	136.48	38.3	/	513			2.33	
PVDF-HFP ENM	Electrospinning	/	108	1380	/	/	/	/	[138]
PVDF-HFP ENM	Electrospinning-*co*-electrospray polystyrene beads	157.6	137	6750	/			/	
PVDF-HFP ENM	Electrospinning	138.5 ± 1.8	131	2433	/	/	/	/	[139]

PVDF-HFP ENM	N-methyl-2-pyrrolidone as the solvent-electrospinning to form beaded nanofiber	153.4 ± 1.1	143	/	/	/	/	/	
		156.6 ± 1.38	149	8235					
		154.1 ± 1.2	146	/					
		155.7 ± 0.7	147	/					
PP FSM	None	128.5 ± 2.5	/	/	/	LW-AB	Water, formamide, and diiodomethane	12.5	[140]
PP FSM	None	118	/	/	/	/	/	/	[106]
PP FSM	None	124.6	33.8	/	/	/	/	/	[3]
PP FSM	None	159 ± 2	103.42	/	/	/	/	/	[104]
PP FSM	None	117	/	/	/	/	/	/	[93]
PP FSM	None	/	160	/	/	/	/	/	[95]
PP FSM	None	110	276.0	/	/	/	/	30	[4]
PP FSM	None	/	/	/	/	/	/	30	[6]
PP FSM	None	139	/	87	108	/	/	/	[141]
PP FSM	None	120	200	/	/	/	/	/	[142]
PP FSM	None	118	/	/	/	/	/	/	[7]
PP FSM	None	141	250	/	/	/	/	/	[60]
PP FSM	None	91	/	/	/	/	/	/	[9]
PP FSM	None	118	/	37.3	/	/	/	/	[143]
PP FSM	None	113 ± 1	/	/	125 ± 3	OWRK	Water and glycerinum	51.2 ± 0.4	[11]
PP FSM	None	113 ± 1	/	/	55 ± 3	OWRK	Water, glycerinum, and diiodomethane	51.2 ± 0.4	[103]
PP FSM	None	140 ± 2	/	/	124.87	OWRK	Water and glycerinum	26.7 ± 1.1	[42]
		124 ± 2			95.99			33.5 ± 1.4	
PP FSM	Attaching SiNPs	154	/	126	160	/	/	/	[141]

PP FSM	Attaching SiNPs-fluorination	159	/	82.1	/	/	/	/	[143]
PP HFM	None	/	320	/	/	/	/	30.1	[69]
PP HFM	None	/	140	/	/	/	/	/	[61]
PES FSM	None	123	323.0	/	/	/	/	46	[4]
PES FSM	None	/	137	/	/	/	/	/	[8]
PES FSM	None	131	290	/	/	/	/	/	[101]
PES FSM	None	132	400	/	/	/	/	23.7	[144]
PES FSM	None	132	390	/	/	/	/	/	[60]
		129	280						
		159	230						
		118	340						
		126	70						
PES FSM	Attaching fluorinated SiNPs	157	260.8	226	/	/	/	/	[145]
PE FSM	None	138	390	/	/	/	/	31.2	[144]
PE FSM	None	/	/	/	/	/	/	33.2	[6]
PE FSM	None	120	300	/	/	/	/	/	[101]
PE FSM	None	120	390	/	/	/	/	/	[60]
PE FSM	None	118	>380	/	/	/	/	/	[99]
		>118	>400						
PE FSM	None	83.2	50	34.3	47.5	/	/	/	[146]
		106.4	60	54.5	67.6				
		108.3	70	62.2	75.8				
		85.3	70	/	/				
PE FSM	Micromolding phase inversion	154.6	/	/	/	/	/	/	[147]
PE HFM	None	/	420	/	/	/	/	30.3	[69]

PSf FSM	None	131.8	/	/	/	/	/	/	[148]
PSf FSM	None	/	/	/	/	/	/	41.0	[6]
PSf FSM	None	116<br>94.3	/	/	/	/	/	/	[9]
PSf FSM	Attaching SiNPs-fluorination	156.6	/	/	/	/	/	/	[148]
PSf HFM	None	82.8	/	/	/	/	/	/	[149]
PAN FSM	Fluorination	132	> 700	42.6	/	LW-AB	Water, glycerinum, and diiodomethane	9.89	[102]
Cellulose acetate ENM	Electrospinning-fluorination	147.5 ± 1.3	/	338	425	LW-AB	Water, glycerinum, and diiodomethane	8.67	[14]
Cellulose acetate ENM	Incorporating SiNPs-electrospinning-fluorination	155.6 ± 3.9	/	520	673			1.56	
Quartz fiber membrane	Coating PDVF-HFP and trimethoxy(propyl)silane	133.75	28.5 ± 0.5	/	/	Wu's harmonic mean	Water and diiodomethane	46.28	[150]
Quartz fiber membrane	Coating PDVF-HFP and fluoroalkylsilanes	151.20	39.0 ± 0.8	/	/			19.56	
Ceramic FSM	Silanization	131.16	192.52	/	/	OWRK	/	27.01	[151]
Ceramic FSM	Fluorination	136	/	6.94	/	OWRK	Water and glycerinum	22.3	[152]
Ceramic FSM	Silanization	135 ± 1	/	/	23.2 ± 1.2	OWRK	Water, glycerinum, and diiodomethane	24.8 ± 0.4	[103]
Ceramic	Fluorination	142 ± 1	/	/	18.1 ±			15.7 ± 0.3	

FSM					0.8				
Ceramic HFM	Fluorination	142 ± 3	260	222	/			/	
Ceramic HFM	Attaching SiNPs-fluorination	161 ± 2	523	464	/	/	/	/	[153]
Ceramic HFM	Attaching SiNPs-fluorination	167 ± 2	540	542	/			/	
Ceramic HFM	Fluorination	131.9 ± 1.1	/	51.4	/			/	
Ceramic HFM	Attaching TiO_2 nanorods-fluorination	155.9 ± 2.5	/	87.8	/	/	/	/	[154]
Ceramic HFM	Attaching TiO_2 microflowers-fluorination	161.6 ± 3.3	/	136.8	/			/	
Ceramic tubular membrane	Silanization	158	/	/	/	LW-AB	Water, ethylene glycol, and diiodomethane	5.1 ± 1.3	[155]
Glass fiber membrane	Attaching ZnO nanoparticles-fluorination	152.8 ± 1.1	/	/	/	/	/	/	[156]
Glass fiber membrane	Fluorination	142.67	/	/	/	/	/	/	[157]
Glass fiber membrane	Attaching SiNPs-fluorination	153.42	/	/	/			/	
Quartz fiber membrane	Attaching SiNPs-fluorination	151.5 ± 4.1	/	/	/	/	/	/	[158]

a) Ra and Rq refer to the arithmetic average roughness and the root mean square average roughness, respectively.

b) FSM: flat sheet membrane.

c) HFM: hollow fiber membrane.

d) ENM: electrospun nanofiber membrane.

Table S2. One way analysis of variance (ANOVA) of **Figure 2**.

Figure 2a

Descriptive statistics

	N analysis	N missing	Mean	Standard deviation	SE of mean
PVDF	73	0	113.01068	18.71179	2.19005
PTFE	56	0	131.05714	11.91779	1.59258
PVDF-HFP	15	0	114.17533	14.90423	3.84825
PP	16	0	123.38125	15.72574	3.93144
PES	8	0	131.25	12.23286	4.32497
PE	9	0	110.8	17.5015	5.83383

One way ANOVA

	DF	Sum of squares	Mean square	F value	Prob > F
Model	5	12839.9214	2567.98428	10.13243	1.62911E-8
Error	171	43338.60456	253.44213		
Total	176	56178.52596			

Null Hypothesis: The means of all levels are equal.

Alternative Hypothesis: The means of one or more levels are different.

At the 0.05 level, the population means are significantly different.

Fit statistics

R-square	Coeff var	Root MSE	Data mean
0.22856	0.13215	15.91987	120.46842

Figure 2b

Descriptive statistics

	N analysis	N missing	Mean	Standard deviation	SE of mean
PVDF	55	0	287.31636	217.68398	29.3525
PTFE	42	0	283.15498	180.37319	27.83219
PVDF-HFP	6	0	289.41167	118.21657	48.26171
PP	8	0	185.4025	94.95972	33.57333
PES	9	0	273.33333	111.07317	37.02439
PE	10	0	253	166.93645	52.78994

One way ANOVA

	DF	Sum of squares	Mean square	F value	Prob > F
Model	5	81285.27764	16257.05553	0.46074	0.8048
Error	124	4.37528E6	35284.51813		
Total	129	4.45657E6			

Null Hypothesis: The means of all levels are equal.

Alternative Hypothesis: The means of one or more levels are different.

At the 0.05 level, the population means are not significantly different.

Fit statistics

R-square	Coeff var	Root MSE	Data mean
0.01824	0.68012	187.84174	276.18922

Figure 2c

Descriptive statistics

	N analysis	N missing	Mean	Standard deviation	SE of mean
PVDF	45	0	32.91311	15.58261	2.32292
PTFE	16	0	22.1075	14.06097	3.51524
PVDF-HFP	4	0	7.4975	1.35837	0.67918

PP	8	0	33.15	12.81127	4.52947
PES	2	0	34.85	15.76848	11.15
PE	3	0	31.56667	1.48436	0.857

One way ANOVA

	DF	Sum of squares	Mean square	F value	Prob > F
Model	5	3511.12453	702.22491	3.35789	0.00879
Error	72	15057.13241	209.12684		
Total	77	18568.25694			

Null Hypothesis: The means of all levels are equal.

Alternative Hypothesis: The means of one or more levels are different.

At the 0.05 level, the population means are significantly different.

Fit statistics

R-square	Coeff var	Root MSE	Data mean
0.18909	0.49162	14.46122	29.41538

Table S3. One way ANOVA of **Figure 4**.

Figure 4a

Descriptive statistics

	N analysis	N missing	Mean	Standard deviation	SE of mean
Pristine	181	0	120.15365	18.01429	1.33899
Micromolding	5	0	152.24	13.3065	5.95085
Electrospinning	56	0	140.06089	10.26169	1.37128
Nanomaterial	48	0	148.16875	18.08382	2.61017

One way ANOVA

	DF	Sum of squares	Mean square	F value	Prob > F

Model	3	41343.32567	13781.10856	49.09398	0
Error	286	80282.68917	280.7087		
Total	289	121626.01484			

Null Hypothesis: The means of all levels are equal.

Alternative Hypothesis: The means of one or more levels are different.

At the 0.05 level, the population means are significantly different.

Fit statistics

R-square	Coeff var	Root MSE	Data mean
0.33992	0.12969	16.75436	129.188

Figure 4b

Descriptive statistics

	N analysis	N missing	Mean	Standard deviation	SE of mean
Pristine	130	0	276.18922	185.86829	16.30173
Micromolding	2	0	234	7.07107	5
Electrospinning	49	0	77.62041	41.73422	5.96203
Nanomaterial	31	0	183.05097	141.57638	25.42787

One way ANOVA

	DF	Sum of squares	Mean square	F value	Prob > F
Model	3	1.44354E6	481179.93644	19.46606	3.66009E-11
Error	208	5.14154E6	24718.92056		
Total	211	6.58508E6			

Null Hypothesis: The means of all levels are equal.

Alternative Hypothesis: The means of one or more levels are different.

At the 0.05 level, the population means are significantly different.

Fit statistics

R-square	Coeff var	Root MSE	Data mean
0.21921	0.72695	157.22252	216.27632

Figure 4c

Descriptive statistics

	N analysis	N missing	Mean	Standard deviation	SE of mean
Pristine	79	0	29.56203	15.48397	1.74208
Micromolding	2	0	45.6	2.40416	1.7
Electrospinning	17	0	26.07706	13.22698	3.20801
Nanomaterial	17	0	14.73176	11.86797	2.87841

One way ANOVA

	DF	Sum of squares	Mean square	F value	Prob > F
Model	3	3781.56913	1260.52304	5.88896	9.09479E-4
Error	111	23759.36668	214.04835		
Total	114	27540.93581			

Null Hypothesis: The means of all levels are equal.

Alternative Hypothesis: The means of one or more levels are different.

At the 0.05 level, the population means are significantly different.

Fit statistics

R-square	Coeff var	Root MSE	Data mean
0.13731	0.5392	14.63039	27.13348

Table S4. One way ANOVA of **Figure 7**.

Figure 7a

Descriptive statistics

	N analysis	N missing	Mean	Standard deviation	SE of mean

Pristine	181	0	120.15365	18.01429	1.33899
Fluorination	19	0	136.66	13.43504	3.08221
Micromolding-Fluorination	6	0	152.81667	16.79433	6.85626
Electrospinning-Fluorination	7	0	151.60143	7.78798	2.94358
Nanomaterial-Fluorination	81	0	155.33333	10.51308	1.16812

One way ANOVA

	DF	Sum of squares	Mean square	F value	Prob > F
Model	4	75171.29492	18792.82373	75.14236	0
Error	289	72277.82321	250.09627		
Total	293	147449.11813			

Null Hypothesis: The means of all levels are equal.

Alternative Hypothesis: The means of one or more levels are different.

At the 0.05 level, the population means are significantly different.

Fit statistics

R-square	Coeff var	Root MSE	Data mean
0.50981	0.11951	15.81443	132.3281

Figure 7b

Descriptive statistics

	N analysis	N missing	Mean	Standard deviation	SE of mean
Pristine	130	0	276.18922	185.86829	16.30173
Fluorination	11	0	249.76364	162.11297	48.8789
Micromolding-Fluorination	1	0	321	/	/
Electrospinning-Fluorination	7	0	161.24286	65.34023	24.69629
Nanomaterial-Fluorination	43	0	330.6193	125.62012	19.15689

One way ANOVA

	DF	Sum of squares	Mean square	F value	Prob > F
Model	4	220904.18175	55226.04544	1.90971	0.11054
Error	187	5.40777E6	28918.53014		
Total	191	5.62867E6			

Null Hypothesis: The means of all levels are equal.

Alternative Hypothesis: The means of one or more levels are different.

At the 0.05 level, the population means are not significantly different.

Fit statistics

R-square	Coeff var	Root MSE	Data mean
0.03925	0.60109	170.05449	282.90796

Figure 7c

Descriptive statistics

	N analysis	N missing	Mean	Standard deviation	SE of mean
Pristine	79	0	29.56203	15.48397	1.74208
Fluorination	10	0	12.77	7.97102	2.52066
Micromolding-Fluorination	1	0	0.27	/	/
Electrospinning-Fluorination	2	0	0.4375	0.09263	0.0655
Nanomaterial-Fluorination	24	0	4.59	5.18986	1.05937

One way ANOVA

	DF	Sum of squares	Mean square	F value	Prob > F
Model	4	14041.82559	3510.4564	19.58871	3.20755E-12
Error	111	19892.10086	179.20812		
Total	115	33933.92644			

Null Hypothesis: The means of all levels are equal.

Alternative Hypothesis: The means of one or more levels are different.

At the 0.05 level, the population means are significantly different.			
Fit statistics			
R-square	Coeff var	Root MSE	Data mean
0.4138	0.6032	13.38686	22.19315

Table S5. Estimated log BAF, log BCF, and log K_{ow} values for organofluorides.[55]

Organofluorides	log BAF[a)]	log BCF[b)]	log K_{ow}	Ref.
1H,1H,2H,2H-perfluorotetradecyltriethoxysilane (FC12)	4.6	2.1	11.1	[159]
1H,1H,2H,2H-perfluorododecyltriethoxysilane (FC10)	5.8	2.8	9.72	[159]
(heptadecafluoro-1,1,2,2-tetrahydrodecyl)triethoxysilane (FC8)	6.7	3.5	8.4	[29]
1H,1H,2H,2H-perfluorodecyltriethoxysilane (FC6)	6.7	3.4	8.3	[159]
Nonafluorohexyltriethoxysilane (FC4)	4.7	3.4	5.7	[29]
(3,3,3-trifluoropropyl)triethoxysilane (FC1)	2.3	1.9	3.4	[29]

a) log BAF values are calculated by the Arnot-Gobas method (upper trophic).[160]

b) log BCF values are computed by a regression-based method.[161]

Table S6. Tests of various hydrophobic membranes in the DCMD process[a)].

Feed solutions	Membrane types	Enhanced methods	Feed temperature (℃)	Permeate temperature (℃)	Initial flux (L m^{-2} h^{-1})	Final flux (L m^{-2} h^{-1})	Initial conductivity (μs cm^{-1})	Final conductivity (μs cm^{-1})	Initial salt rejection (%)	Final salt rejection (%)	Ref.
3.5 wt% NaCl	PVDF FSM	None	65	20	30	30@1 h	/	/	99.97	99.90@2 h	[148]
3.5 wt% NaCl	PVDF FSM	None	60	20	17	17@50 h	1.2	1.2@50 h	/	/	[84]

Feed solutions	Membrane types	Enhanced methods	Feed temperature (℃)	Permeate temperature (℃)	Initial flux (L m^{-2} h^{-1})	Final flux (L m^{-2} h^{-1})	Initial conductivity (μs cm^{-1})	Final conductivity (μs cm^{-1})	Initial salt rejection (%)	Final salt rejection (%)	Ref.
3.5 wt% NaCl	PVDF FSM	None	60	20	20.24	20.24@24 h	1.74	1.74@24 h	/	/	[79]
3.5 wt% NaCl	PVDF FSM	None	60	20	31.9	31.9@20 h	6.6	10@20 h	/	/	[128]
3.5 wt% NaCl	PVDF FSM	None	70	20	29.2	29.2@24 h	6.4	6.4@24 h	/	/	[17]
3.5 wt% NaCl	PVDF FSM	None	60	20	21	21@10 h	2.5	2.5@10 h	/	/	[74]
3.5 wt% NaCl	PVDF FSM	None	53	20	8.2	8.2@30 h	/	/	/	/	[14]
3.5 wt% NaCl	PVDF FSM	None	60	20	10	8.1@24 h	2.4	2.4@24 h	/	/	[82]
3.5 wt% NaCl	PTFE FSM	None	53	20	10.8	10.8@30 h	/	/	/	/	[14]
3.5% NaCl	PVDF FSM	None	60	20	30.5	30.5@2 h	/	/	100	100@2 h	[54]
35 g L^{-1} NaCl	PVDF FSM	None	70	20	37	37@72 h	1.56	7@72 h	/	/	[44]
35 g L^{-1} NaCl	PVDF FSM	None	65	15	29.9	29@72 h	1.56	5@72 h	/	/	[31]
35 g L^{-1} NaCl	PTFE FSM	None	53	20	20	20@25 h	/	/	/	/	[88]

Feed solutions	Membrane types	Enhanced methods	Feed temperature (℃)	Permeate temperature (℃)	Initial flux (L m^{-2} h^{-1})	Final flux (L m^{-2} h^{-1})	Initial conductivity (μs cm^{-1})	Final conductivity (μs cm^{-1})	Initial salt rejection (%)	Final salt rejection (%)	Ref.
4 wt% NaCl	PVDF FSM	None	60	20	20.3	20.3@10 h	2.29	3.2@10 h	/	/	[12]
4% NaCl	PVDF FSM	None	60	20	21.45	21.45@1.6 h	/	/	/	/	[25]
1 M NaCl	PVDF FSM	None	60	20	22.5	22.5@1.5 h	/	/	100	100@1.5 h	[162]
1 M NaCl	PVDF FSM	None	60	20	27	27@1 h	/	/	100	100@1 h	[29]
14.7 mM $CaSO_4$	PVDF FSM	None	70	20	35.1	17.2	/	/	/	/	[20]
20 mM Na_2SO_4 and 20 mM $CaCl_2$	PVDF FSM	None	60	20	21	7@8 h	3.4	15.5@6 h	/	/	[74]
20 mM Na_2SO_4 and 20 mM $CaCl_2$	PVDF FSM	None	60	20	30.5	12.2@23.33 h	0	306@23.33 h	/	/	[54]
3.5 wt% NaCl and 50~200 ppm HA[b)]	PVDF FSM	None	70	20	30	24.6@6 h	/	/	100	98@6 h	[17]
35 g L^{-1} NaCl and 50 mg L^{-1} HA	PVDF FSM	None	65	15	29.9	21@75 h	1.56	125.12@75 h	/	/	[31]

Feed solutions	Membrane types	Enhanced methods	Feed temperature (℃)	Permeate temperature (℃)	Initial flux (L m^{-2} h^{-1})	Final flux (L m^{-2} h^{-1})	Initial conductivity (μs cm^{-1})	Final conductivity (μs cm^{-1})	Initial salt rejection (%)	Final salt rejection (%)	Ref.
3.5 wt% NaCl and 12 mg L^{-1} SDS[c)]	PVDF FSM	None	65	20	30	30@1 h	/	/	99.95	89.97@2 h	[148]
3.5 wt% NaCl and 0.1 mM SDS	PVDF FSM	None	60	20	19.57	1.7@1.27 h	/	/	100	100@1.27 h	[79]
3.5% NaCl and 0.1 mM SDS	PVDF FSM	None	60	20	20.7	9.73@4 h	/	/	100	99.97@4 h	[163]
3.5 wt% NaCl and 0.1 mM CTAB[d)]	PVDF FSM	None	60	20	14.59	1.29@0.3 h	/	/	100	100@0.3 h	[79]
3.5% NaCl and 0.1~0.2 mM SDS	PVDF FSM	None	60	20	/	/	/	/	100	76@2 h	[1]
3.5% NaCl and 0.1~0.3 mM SDS	PVDF FSM	None	60	20	/	/	/	/	100	45@3 h	[16]
3.5 wt% NaCl and 0.1~0.3 mM SLS[e)]	PVDF FSM	None	60	20	16.88	12.62@6 h	/	/	100	100@6 h	[79]
3.5 wt% NaCl and 0.1~0.3 mM	PVDF FSM	None	53	20	/	/	/	/	100	30@6 h	[14]

Feed solutions	Membrane types	Enhanced methods	Feed temperature (℃)	Permeate temperature (℃)	Initial flux (L m^{-2} h^{-1})	Final flux (L m^{-2} h^{-1})	Initial conductivity (μs cm^{-1})	Final conductivity (μs cm^{-1})	Initial salt rejection (%)	Final salt rejection (%)	Ref.
SDS											
3.5 wt% NaCl and 0.1~0.3 mM SDS	PTFE FSM	None	53	20	/	/	/	/	100	44.6@6 h	[14]
3.5 wt% NaCl and 0.1~0.4 mM SDS	PVDF FSM	None	70	20	30	9.9@6 h	/	/	100	36.4@6 h	[17]
3.5% NaCl and 0.1~0.4 mM SDS	PVDF FSM	None	60	20	/	/	/	/	100	40.5@7.67 h	[22]
3.5 wt% NaCl and 0.1~0.4 mM DTAC[f)]	PVDF FSM	None	70	20	30	9@6 h	/	/	100	~30@6 h	[17]
3.5 wt% NaCl and 0.1~0.5 mM DTAB[g)]	PVDF FSM	None	60	20	20	17@10 h	/	/	100	100@10 h	[79]
1 M NaCl and 0.1~0.2 mM SDS	PVDF FSM	None	60	20	/	/	/	/	100	0@1.5 h	[162]
3.5% NaCl, 5 mg L^{-1} AOM, and 0.1~0.2 mM	PVDF FSM	None	60	20	22.9	6.87@1 h	/	/	100	71.3@1.66 h	[1]

Feed solutions	Membrane types	Enhanced methods	Feed temperature (°C)	Permeate temperature (°C)	Initial flux (L m^{-2} h^{-1})	Final flux (L m^{-2} h^{-1})	Initial conductivity (μs cm^{-1})	Final conductivity (μs cm^{-1})	Initial salt rejection (%)	Final salt rejection (%)	Ref.
SDS											
35 g L^{-1} NaCl, 50 mg L^{-1} HA, and 0.1~0.4 mM SDS	PVDF FSM	None	65	15	29.9	6.8@16 h	0	4280@16 h	/	/	[31]
35 g L^{-1} NaCl, 1.26 g L^{-1} $CaCl_2$, 10 mg L^{-1} HA, and 10 mg L^{-1} SDBS$^{h)}$	PVDF FSM	None	70	20	38.1	18@150 h	1.56	166.5@150 h	/	/	[44]
100 g L^{-1} NaCl, 30 g L^{-1} $MgSO_4$, 1.26 g L^{-1} $CaCl_2$, 10 mg L^{-1} HA, and 10 mg L^{-1} SDBS	PVDF FSM	None	70	20	38.1	10.8@90 h	1.56	194@90 h	/	/	[44]
3.5 wt % NaCl and 0.005% v/v oil	PVDF FSM	None	60	20	26.13	13.9@2 h	/	/	100	99.8@2 h	[22]
3.5 wt % NaCl and 0.001%~0.01%	PVDF FSM	None	70	20	30	9@6 h	/	/	100	40@6 h	[17]

Feed solutions	Membrane types	Enhanced methods	Feed temperature (℃)	Permeate temperature (℃)	Initial flux (L m-2 h-1)	Final flux (L m-2 h-1)	Initial conductivity (μs cm-1)	Final conductivity (μs cm-1)	Initial salt rejection (%)	Final salt rejection (%)	Ref.
v/v mineral oil 3.5 wt % NaCl and 0.001%~0.01% v/v kerosene	PVDF FSM	None	70	20	30	7.5@6 h	/	/	100	46@6 h	[17]
3.5 wt% NaCl and 10 mg L^{-1} oil	PVDF FSM	None	60	20	14	5.5@3.5 h	4.8	522.2@3.5 h	/	/	[84]
35 g L^{-1} NaCl and 1000 mg L^{-1} crude oil	PTFE FSM	None	53	20	20	0@11 h	/	/	/	/	[88]
3.5 wt% NaCl and 150 ppm DTAB stabilized mineral oil	PVDF FSM	None	60	20	20	13.5@12 h	1.74	1.74@12 h	/	/	[79]
1 M NaCl, 0.05 mM SDS, and 50 ppm oil	PVDF FSM	None	60	20	22.5	2.25@2 h	/	/	100	100@2 h	[162]
Seawater (Hong Kong shoreline)	PVDF FSM	None	60	20	19.5	11.1@50 h	/	/	100	99.8@50 h	[27]
Seawater (East China Sea)	PVDF FSM	None	60	20	28.9	23.85@50 h	0	3.4@50 h	/	/	[164]

Feed solutions	Membrane types	Enhanced methods	Feed temperature (℃)	Permeate temperature (℃)	Initial flux (L m-2 h-1)	Final flux (L m-2 h-1)	Initial conductivity (μs cm-1)	Final conductivity (μs cm-1)	Initial salt rejection (%)	Final salt rejection (%)	Ref.
Mimetic AOM[i)] contaminated Seawater	PVDF FSM	None	65	18	21.22	14.2@24 h	0	200.3@24 h	100	96.8@24 h	[52]
Seawater (Hong Kong shoreline) and 0.1 mM SDS	PVDF FSM	None	60	20	/	/	/	/	100	45@1.5 h	[27]
Biologically pre-treated coking wastewater	PVDF FSM	None	60	20	25	8.68@120 h	10	30@57 h	/	/	[41]
Imitative oil/gas production waste emulsion	PVDF FSM	None	60	20	10.68	0.25@16.5 h	17.35	601.8@16.5 h	/	/	[25]
3.5 wt% NaCl	PVDF-CTFE FSM	Micromolding phase inversion	70	25	16.3	15.4@48 h	/	/	100	100@48 h	[67]
3.5 wt% NaCl	PE FSM	Micromolding phase inversion	60	20	21.8	21.8@50 h	3.3	4.8@50 h	/	/	[147]
4 wt% NaCl	PVDF FSM	Micromolding phase inversion	60	20	19.9	19.9@10 h	3.4	3.8@10 h	/	/	[12]
14.7 mM $CaSO_4$	PVDF	Micromolding	70	20	31.5	16.7	/	/	/	/	[20]

Feed solutions	Membrane types	Enhanced methods	Feed temperature (°C)	Permeate temperature (°C)	Initial flux (L m-2 h-1)	Final flux (L m-2 h-1)	Initial conductivity (μs cm-1)	Final conductivity (μs cm-1)	Initial salt rejection (%)	Final salt rejection (%)	Ref.
	FSM	phase inversion									
3.5 wt% NaCl, 0.35 g L^{-1} $CaCl_2$, and 0.73 g L^{-1} $NaHCO_3$	PE FSM	Micromolding phase inversion	60	20	20	6@83 h	2.5	5.3@83 h	/	/	[147]
Seawater (Hong Kong shoreline)	PVDF FSM	Micromolding phase inversion	60	20	23	18.8@133 h	/	/	100	99.5@133 h	[27]
Seawater (Hong Kong shoreline) and 0.1 mM SDS	PVDF FSM	Micromolding phase inversion	60	20	23	23@1.5 h	/	/	100	100@1.5 h	[27]
Simulated methyl orange dyeing wastewater	PE FSM	Micromolding phase inversion	60	20	21.4	21.4@50 h	3.2	5.6@50 h	/	/	[147]
Simulated methylene blue dyeing wastewater	PE FSM	Micromolding phase inversion	60	20	19.9	19.9@15 h	3.2	13.7@15 h	/	/	[147]
3.5 wt% NaCl	PVDF ENM	Electrospinning	50	10	17	10.2@100 h	2	37@100 h	/	/	[75]
3.5 wt% NaCl	PVDF	Electrospinning	60	20	25.88	25.88@24 h	1.74	1.74@24 h	/	/	[79]

Feed solutions	Membrane types	Enhanced methods	Feed temperature (℃)	Permeate temperature (℃)	Initial flux (L m^{-2} h^{-1})	Final flux (L m^{-2} h^{-1})	Initial conductivity (μs cm^{-1})	Final conductivity (μs cm^{-1})	Initial salt rejection (%)	Final salt rejection (%)	Ref.
3.5 wt% NaCl	ENM PVDF ENM	Electrospinning	60	20	32	16@10 h	1.7	1.7@10 h	/	/	[74]
3.5 wt% NaCl	PVDF ENM	Electrospinning	60	20	30.5	30.5@50 h	0	97@50 h	/	/	[84]
3.5 wt% NaCl	PVDF ENM	Electrospinning	50	20	49.3	24.65@74 h	9	10@74 h	/	/	[71]
3.5 wt% NaCl	PVDF-HFP ENM	Electrospinning	60	20	51.3	44.2@20 h	5.11	14.44@20 h	/	/	[128]
3.5 wt% NaCl	PVDF-HFP ENM	Electrospinning	60	20	8.98	19.6@72 h	/	/	100	96.7@72 h	[129]
3.5 wt% NaCl	PVDF-HFP ENM	Electrospinning	60	20	29.17	30.8@2 h	/	/	100	~100@2 h	[131]
35 g L^{-1} NaCl	PVDF-HFP ENM	Electrospinning	60	20	31.67	21.2@40 h	/	/	100	99.95@40 h	[139]
35 g L^{-1} NaCl	PVDF-HFP ENM	Electrospinning	60	20	22.6	22.6@1 h	/	/	100	100@1 h	[134]

Feed solutions	Membrane types	Enhanced methods	Feed temperature (°C)	Permeate temperature (°C)	Initial flux (L m^{-2} h^{-1})	Final flux (L m^{-2} h^{-1})	Initial conductivity (μs cm^{-1})	Final conductivity (μs cm^{-1})	Initial salt rejection (%)	Final salt rejection (%)	Ref.
0.6 M NaCl	PVDF ENM	Electrospinning	60	10	32	32@1 h	/	/	100	100@1 h	[81]
1 M NaCl	PVDF-HFP ENM	Electrospinning	60	20	22.28	22.28@2 h	/	/	100	99.8@2 h	[132]
20 mM Na_2SO_4 and 20 mM $CaCl_2$	PVDF ENM	Electrospinning	60	20	38.7	2.5@8 h	0	15.5@6 h	/	/	[74]
30 mM Na_2SO_4 and 30 mM $CaCl_2$	PVDF ENM	Electrospinning	60	20	13	6.24@10 h	3.1	45@10 h	100	99.55@10 h	[78]
3.5 wt% NaCl, 40 mM $NaSO_4$, and 40 mM $CaCl_2$	PVDF-HFP ENM	Electrospinning	60	20	30	11.6@1.2 h	/	/	100	62@1.2 h	[131]
3.5% NaCl, 10 mg L^{-1} HA, 15 mg L^{-1} SA, and 7 mg L^{-1} BSA	PVDF-HFP ENM	Electrospinning	60	20	33.5	13.4@24 h	1	52.23@24 h	/	/	[135]
3.5 wt% NaCl and 0.05~0.1 mM SDS	PVDF ENM	Electrospinning	60	20	13.5	0.07@3.7 h	3	358.5@3.7 h	/	/	[78]

Feed solutions	Membrane types	Enhanced methods	Feed temperature (°C)	Permeate temperature (°C)	Initial flux (L m^{-2} h^{-1})	Final flux (L m^{-2} h^{-1})	Initial conductivity (μs cm^{-1})	Final conductivity (μs cm^{-1})	Initial salt rejection (%)	Final salt rejection (%)	Ref.
3.5 wt% NaCl and 0.1 mM SDS	PVDF ENM	Electrospinning	60	20	17.15	0@0.5 h	/	/	100	100@2 h	[79]
3.5 wt% NaCl and 0.1 mM SDS	PVDF-HFP ENM	Electrospinning	60	20	14.6	14.6@2 h	/	/	100	98@2 h	[138]
3.5% NaCl and 0.1 mM SDS	PVDF-HFP ENM	Electrospinning	60	20	37.2	16.85@4 h	/	/	100	98.1@4 h	[163]
3.5 wt% NaCl and 0.1 mM CTAB	PVDF ENM	Electrospinning	60	20	26	0@0.5 h	/	/	100	100@0.5 h	[79]
3.5% NaCl and 0.1~0.2 mM SDS	PVDF-HFP ENM	Electrospinning	60	20	36.2	22.44@2 h	/	/	100	94@2 h	[1]
3.5 wt% NaCl and 0.1~0.2 mM SLS	PVDF ENM	Electrospinning	60	20	26	5@3.25 h	/	/	100	100@3.25 h	[79]
3.5 wt% NaCl and 0.4 mM SDS	PVDF-HFP ENM	Electrospinning	60	10	12.1	1.4@0.07 h	2.6	11.9@0.07 h	/	/	[136]
3.5 wt% NaCl	PVDF	Electrospinning	60	20	26	10.92@9 h	/	/	100	99.97@9 h	[79]

Feed solutions	Membrane types	Enhanced methods	Feed temperature (℃)	Permeate temperature (℃)	Initial flux (L m^{-2} h^{-1})	Final flux (L m^{-2} h^{-1})	Initial conductivity (μs cm^{-1})	Final conductivity (μs cm^{-1})	Initial salt rejection (%)	Final salt rejection (%)	Ref.
and 0.1~0.5 mM DTAB	ENM										
3.5% NaCl, 5 mg L^{-1} AOM, and 0.1~0.2 mM SDS	PVDF-HFP ENM	Electrospinning	60	20	36.2	12.3@2 h	/	/	100	91@2 h	[1]
3.5 wt% NaCl and 150 ppm DTAB stabilized mineral oil	PVDF ENM	Electrospinning	60	20	26	8.6@5 h	1.74	6@6 h	/	/	[79]
Brackish water (Antwerp)	PVDF ENM	Electrospinning	60	20	12.9	3.5@50 h	/	/	100	94.5@50 h	[85]
Brackish water (Scheldt Estuary)	PVDF ENM	Electrospinning	60	20	13.6	7@50 h	/	/	100	97.7@50 h	[85]
Mimetic AOM contaminated Seawater	PVDF-HFP ENM	Electrospinning	65	18	33.26	22.95@24 h	0	124.8@24 h	100	97.2@24 h	[52]
3.5 wt% NaCl	PVDF ENM	Attaching nanomaterials	50	10	25	25@100 h	2	<5@100 h	/	/	[75]
3.5 wt% NaCl	PVDF ENM	Attaching nanomaterials	60	20	28.4	28.4@50 h	1	1@50 h	/	/	[84]

Feed solutions	Membrane types	Enhanced methods	Feed temperature (°C)	Permeate temperature (°C)	Initial flux (L m^{-2} h^{-1})	Final flux (L m^{-2} h^{-1})	Initial conductivity (μs cm^{-1})	Final conductivity (μs cm^{-1})	Initial salt rejection (%)	Final salt rejection (%)	Ref.
3.5 wt% NaCl	PVDF-HFP ENM	Attaching nanomaterials	60	20	10.45	10.45@720 h	/	/	100	99.99@720 h	[129]
35 g L^{-1} NaCl	PVDF-HFP ENM	Attaching nanomaterials	60	20	40	33.9@40 h	/	/	100	99.95@40 h	[139]
100 g L^{-1} NaCl	PVDF ENM	Attaching nanomaterials	55	20	10.9	10.9@20 h	2	2@20 h	/	/	[87]
3.5 wt% NaCl and 10 mg L^{-1} HA	PVDF ENM	Attaching nanomaterials	60	20	30	30@12 h	1	1@12 h	>99.99	>99.99@12 h	[84]
3.5 wt% NaCl and 10 mg L^{-1} methyl orange	PVDF ENM	Attaching nanomaterials	60	20	28.3	28.3@12 h	1	1@12 h	>99.99	>99.99@12 h	[84]
3.5 wt% NaCl and 10 mg L^{-1} methylene blue	PVDF ENM	Attaching nanomaterials	60	20	29.3	29.3@12 h	1	1@12 h	>99.99	>99.99@12 h	[84]
3.5% NaCl, 10 mg L^{-1} HA, 15 mg L^{-1} SA, and 7 mg L^{-1} BSA	PVDF-HFP ENM	Attaching nanomaterials	60	20	35<br>36.3	18.7@24 h<br>26.8@24 h	1<br>1	3@24 h<br>1.9@24 h	/	/	[135]
3.5% NaCl and	PVDF	Attaching	60	20	20	~20@40 h	/	/	100	97@40 h	[39]

Feed solutions	Membrane types	Enhanced methods	Feed temperature (°C)	Permeate temperature (°C)	Initial flux (L m^{-2} h^{-1})	Final flux (L m^{-2} h^{-1})	Initial conductivity (μs cm^{-1})	Final conductivity (μs cm^{-1})	Initial salt rejection (%)	Final salt rejection (%)	Ref.
0.1 mM SDS	FSM	nanomaterials									
3.5% NaCl and 0.1 mM SDS	PVDF FSM	Attaching nanomaterials	60	20	~22	~22@200 h	/	/	100	>99.9@200 h	[16]
3.5% NaCl and 0.1 mM SDS	PVDF-HFP ENM	Attaching nanomaterials	60	20	31.5	27.9@4 h	/	/	100	99.94@4 h	[163]
3.5 wt% NaCl and 50 mg L^{-1} SDBS	PVDF ENM	Attaching nanomaterials	60	20	28.6	28.6@12 h	1	1@12 h	>99.99	>99.99@12 h	[84]
3.5 wt% NaCl and 0.1~0.2 mM SDS	PVDF-HFP ENM	Attaching nanomaterials	60	20	13.4	13.4@4 h	/	/	100	99@4 h	[138]
3.5% NaCl and 0.1~0.3 mM SDS	PVDF FSM	Attaching nanomaterials	60	20	22	21.9@4.5 h	/	/	100	100@4.5 h	[16]
3.5% NaCl and 0.1~0.5 mM SDS	PVDF-HFP ENM	Attaching nanomaterials	60	20	33.1	27.8@5 h	/	/	100	99@5 h	[1]
3.5% NaCl, 5 mg L^{-1} AOM, and 0.1~0.5 mM	PVDF-HFP ENM	Attaching nanomaterials	60	20	33.1	20.5@5 h	/	/	100	95@5 h	[1]

Feed solutions	Membrane types	Enhanced methods	Feed temperature (℃)	Permeate temperature (℃)	Initial flux (L m^{-2} h^{-1})	Final flux (L m^{-2} h^{-1})	Initial conductivity (μs cm^{-1})	Final conductivity (μs cm^{-1})	Initial salt rejection (%)	Final salt rejection (%)	Ref.
SDS<br>3.5 wt% NaCl and 10 mg L^{-1} oil	PVDF ENM	Attaching nanomaterials	60	20	28.4	28.4@12 h	1	1@12 h	>99.99	>99.99@12 h	[84]
Brackish water (Antwerp)	PVDF ENM	Attaching nanomaterials	60	20	15.6<br>36.4	3.9@50 h<br>13.9@50 h	/	/	100<br>100	94.2@50 h<br>93.6@50 h	[85]
Brackish water (Scheldt Estuary)	PVDF ENM	Attaching nanomaterials	60	20	14.2<br>37.1	5.7@50 h<br>21.5@50 h	/	/	100<br>100	96.9@50 h<br>95.4@50 h	[85]
4% NaCl	PVDF FSM	Fluorination	60	20	8.54	8.54@1.6 h	/	/	/	/	[25]
1 M NaCl	Glass fiber membrane	Fluorination	60	20	20.6	20.6@2 h	/	/	100	~100@2 h	[157]
1 M NaCl	Glass fiber membrane	Fluorination	60	20	12.5	12.3@2 h	/	/	100	~100@2 h	[156]
3.5 wt% NaCl and 0.1~0.4 mM SDS	PVDF FSM	Fluorination	70	20	27.5	23.9@6 h	/	/	100	97.6@6 h	[17]

Feed solutions	Membrane types	Enhanced methods	Feed temperature (°C)	Permeate temperature (°C)	Initial flux (L m-2 h-1)	Final flux (L m-2 h-1)	Initial conductivity (μs cm-1)	Final conductivity (μs cm-1)	Initial salt rejection (%)	Final salt rejection (%)	Ref.
1 M NaCl and 0.1~0.3 mM SDS	Glass fiber membrane	Fluorination	60	20	12.5	2.1@6 h	/	/	100	~100@6 h	[156]
Imitative oil/gas production waste emulsion	PVDF FSM	Fluorination	60	20	10	0.25@16.5 h	17.35	359.8@16.5 h	/	/	[25]
3.5 wt% NaCl	PVDF-CTFE FSM	Micromolding phase inversion-Fluorination	70	25	14.2	13.4@132 h	/	/	100	100@132 h	[67]
4 wt% NaCl	PVDF FSM	Micromolding phase inversion-Fluorination	60	20	20	20@10 h	2.6	2.6@10 h	/	/	[12]
14.7 mM $CaSO_4$	PVDF FSM	Micromolding phase inversion-Fluorination	70	20	24.3	22.6	/	/	/	/	[20]
Seawater (Hong Kong shoreline)	PVDF FSM	Micromolding phase inversion-	60	20	23.5	19@133 h	/	/	100	99.7@133 h	[27]

Feed solutions	Membrane types	Enhanced methods	Feed temperature (°C)	Permeate temperature (°C)	Initial flux (L m^{-2} h^{-1})	Final flux (L m^{-2} h^{-1})	Initial conductivity (μs cm^{-1})	Final conductivity (μs cm^{-1})	Initial salt rejection (%)	Final salt rejection (%)	Ref.
Seawater (Hong Kong shoreline) and 0.1~0.4 mM SDS	PVDF FSM	Fluorination Micromolding phase inversion-Fluorination	60	20	23.5	23.5@6 h	/	/	100	100@6 h	[27]
3.5 wt% NaCl	PVDF ENM	Electrospinning -Fluorination	60	20	25.9	25.9@24 h	~2.5	~2.5@24 h	/	/	[82]
3.5 wt% NaCl	Cellulose acetate ENM	Electrospinning -Fluorination	53	20	12.3	12.3@30 h	/	/	/	/	[14]
3.5 wt% NaCl and 0.05~0.1 mM SDS	PVDF ENM	Electrospinning -Fluorination	60	20	25.9	25.9@4 h	/	/	100	100@4 h	[82]
0.6 M NaCl and 0.4 mM SDS	PVDF ENM	Electrospinning -Fluorination	60	10	25.9	25.9@90 h	/	/	100	100@90 h	[81]
3.5 wt% NaCl and 0.4 mM SDS	PVDF-HFP ENM	Electrospinning -Fluorination	60	10	10.5	10.5@8 h	1	1@8 h	/	/	[136]
20 mM $CaSO_4$ and 0.4 mM SDS	PVDF ENM	Electrospinning -Fluorination	60	10	25.5	7.65@33.33 h	1.5	3.8@33.33 h	/	/	[81]
3.5 wt% NaCl	PVDF	Attaching	70	20	27.7	27.7@24 h	5	5.8@24 h	/	/	[17]

Feed solutions	Membrane types	Enhanced methods	Feed temperature (°C)	Permeate temperature (°C)	Initial flux (L m^{-2} h^{-1})	Final flux (L m^{-2} h^{-1})	Initial conductivity (μs cm^{-1})	Final conductivity (μs cm^{-1})	Initial salt rejection (%)	Final salt rejection (%)	Ref.
	FSM	nanomaterials-Fluorination									
3.5 wt% NaCl	PSf FSM	Attaching nanomaterials-Fluorination	65	20	5	5@1 h	/	/	99.99	99.99@2 h	[148]
3.5 wt% NaCl	PVDF ENM	Attaching nanomaterials-Fluorination	60	20	23.76	23.76@24 h	1.74	1.74@24 h	/	/	[79]
3.5 wt% NaCl	PVDF ENM	Attaching nanomaterials-Fluorination	60	20	37	37@10 h	2.5	2.8@10 h	/	/	[74]
3.5 wt% NaCl	PVDF-HFP ENM	Attaching nanomaterials-Fluorination	60	20	45	42.5@20 h	5	7@20 h	/	/	[128]
3.5 wt% NaCl	Cellulose acetate ENM	Attaching nanomaterials-Fluorination	53	20	13.6	13.6@30 h	/	/		/	[14]
3.5% NaCl	PVDF FSM	Attaching nanomaterials-Fluorination	60	20	24.5	22.05@24 h	0.12	0.12@24 h	/	/	[54]
3.5% NaCl	PVDF ENM	Attaching nanomaterials-	60	15	29.4	29.4@8 h	2	2@8 h	/	/	[80]

Feed solutions	Membrane types	Enhanced methods	Feed temperature (℃)	Permeate temperature (℃)	Initial flux (L m^{-2} h^{-1})	Final flux (L m^{-2} h^{-1})	Initial conductivity (μs cm^{-1})	Final conductivity (μs cm^{-1})	Initial salt rejection (%)	Final salt rejection (%)	Ref.
		Fluorination									
35 g L^{-1} NaCl	PVDF FSM	Attaching nanomaterials-Fluorination	70	20	36.5	36.5@72 h	1.56	2.45@72 h	/	/	[44]
35 g L^{-1} NaCl	PVDF FSM	Attaching nanomaterials-Fluorination	65	15	36.3	36.3@72 h	1.56	1.73@72 h	/	/	[31]
4% NaCl	PVDF FSM	Attaching nanomaterials-Fluorination	60	20	4.84	4.84@1.6 h	/	/		/	[25]
1 M NaCl	PVDF FSM	Attaching nanomaterials-Fluorination	60	20	16.4	16.4@1.5 h	/	/	100	100@1.5 h	[162]
20 mM Na_2SO_4 and 20 mM $CaCl_2$	PVDF FSM	Attaching nanomaterials-Fluorination	60	20	25.5	23.97@23.33 h	0	15.15@23.33 h	/	/	[54]
20 mM Na_2SO_4 and 20 mM $CaCl_2$	PVDF ENM	Attaching nanomaterials-Fluorination	60	20	30	27.5@8 h	0	1.8@8 h	/	/	[74]
30 mM Na_2SO_4 and 30 mM $CaCl_2$	PVDF ENM	Attaching nanomaterials-Fluorination	60	20	15	13.35@10 h	3.1	8@10 h	100	99.9@10 h	[78]

Feed solutions	Membrane types	Enhanced methods	Feed temperature (°C)	Permeate temperature (°C)	Initial flux (L m^{-2} h^{-1})	Final flux (L m^{-2} h^{-1})	Initial conductivity (μs cm^{-1})	Final conductivity (μs cm^{-1})	Initial salt rejection (%)	Final salt rejection (%)	Ref.
3.5 wt% NaCl, 40 mM $NaSO_4$, and 40 mM $CaCl_2$	PVDF-HFP ENM	Attaching nanomaterials-Fluorination	60	20	18.9	18.9@72 h	/	/	100	100@72 h	[131]
3.5 wt% NaCl and 10 mg L^{-1} HA	Ceramic HFM	Attaching nanomaterials-Fluorination	80	10	49.41	43.7@6.67 h	213	213@6.67 h	/	/	[153]
3.5% NaCl and 10 mg L^{-1} HA	PVDF ENM	Attaching nanomaterials-Fluorination	60	15	23.5	23.5@8 h	2	2@8 h	/	/	[80]
3.5 wt% NaCl and 50~200 ppm HA	PVDF FSM	Attaching nanomaterials-Fluorination	70	20	28	~27@6 h	/	/	100	100@6 h	[17]
35 g L^{-1} NaCl and 50 mg L^{-1} HA	PVDF FSM	Attaching nanomaterials-Fluorination	65	15	36.3	36.3@75 h	1.56	1.73@75 h	100	100@75 h	[31]
3.5% NaCl and 10 mg L^{-1} methylene blue	PVDF ENM	Attaching nanomaterials-Fluorination	60	15	26	26@8 h	2	2@8 h	/	/	[80]
5 g L^{-1} NaCl and 0.5 mM SDBS	PES FSM	Attaching nanomaterials-Fluorination	50	20	12.6	10.7@9.3 h	1.9	40@9.3 h	100	99.77@9.3 h	[145]

Feed solutions	Membrane types	Enhanced methods	Feed temperature (℃)	Permeate temperature (℃)	Initial flux (L m^{-2} h^{-1})	Final flux (L m^{-2} h^{-1})	Initial conductivity (μs cm^{-1})	Final conductivity (μs cm^{-1})	Initial salt rejection (%)	Final salt rejection (%)	Ref.
3.5% NaCl and 10 mg L^{-1} SDBS	PVDF ENM	Attaching nanomaterials-Fluorination	60	15	28.2	28.2@8 h	2	2@8 h	/	/	[80]
3.5 wt% NaCl and 12 mg L^{-1} SDS	PSf FSM	Attaching nanomaterials-Fluorination	65	20	5	5@1 h	/	/	99.95	93.87@2 h	[148]
3.5 wt% NaCl and 0.1 mM CTAB	PVDF ENM	Attaching nanomaterials-Fluorination	60	20	17.62	0@0.75 h	/	/	100	100@0.75 h	[79]
3.5 wt% NaCl and 0.1 mM SDS	PVDF FSM	Attaching nanomaterials-Fluorination	60	20	17.3	17.3@216 h	/	/	100	99.99@216 h	[5]
3.5 wt% NaCl and 0.2 mM SDS	Cellulose acetate ENM	Attaching nanomaterials-Fluorination	53	20	13.6	13.6@120 h	7	7@120 h	/	/	[14]
3.5 wt% NaCl and 0.1~0.3 mM SDS	PVDF ENM	Attaching nanomaterials-Fluorination	60	20	23	8.05@6 h		/	100	99.84@6 h	[79]
3.5 wt% NaCl and 0.1~0.3 mM SLS	PVDF ENM	Attaching nanomaterials-Fluorination	60	20	23	14.72@6 h	/	/	100	100@6 h	[79]
3.5 wt% NaCl	PVDF	Attaching	60	20	24.29	20.6@8 h	/	/	100	98.6@8 h	[22]

Feed solutions	Membrane types	Enhanced methods	Feed temperature (℃)	Permeate temperature (℃)	Initial flux (L m^{-2} h^{-1})	Final flux (L m^{-2} h^{-1})	Initial conductivity (μs cm^{-1})	Final conductivity (μs cm^{-1})	Initial salt rejection (%)	Final salt rejection (%)	Ref.
and 0.1~0.4 mM SDS	FSM	nanomaterials-Fluorination									
3.5 wt% NaCl and 0.1~0.4 mM SDS	PVDF FSM	Attaching nanomaterials-Fluorination	70	20	28	~27@6 h	/	/	100	99.99@6 h	[17]
3.5 wt% NaCl and 0.1~0.4 mM DTAC	PVDF FSM	Attaching nanomaterials-Fluorination	70	20	28	~27@6 h	/	/	100	99.99@6 h	[17]
3.5 wt% NaCl and 0.4 mM SDS	PVDF-HFP ENM	Attaching nanomaterials-Fluorination	60	20	17.8	17.8@72 h	/	/	100	100@72 h	[131]
3.5 wt% NaCl and 0.1~0.5 mM SDS	Cellulose acetate ENM	Attaching nanomaterials-Fluorination	53	20	13.6	13.6@10 h	/	/	100	100@10 h	[14]
3.5 wt% NaCl and 0.1~0.5 mM DTAB	PVDF ENM	Attaching nanomaterials-Fluorination	60	20	23	22.54@10 h	/	/	100	100@10 h	[79]
3.5 wt% NaCl and 0.05~0.6 mM SDS	PVDF ENM	Attaching nanomaterials-Fluorination	60	20	14.45	10@9 h	3	32.3@9 h	/	/	[78]
3.5% NaCl and 0.1~2.0 mM	PVDF FSM	Attaching nanomaterials-	60	20	25.5	20.9@26 h	/	/	100	99@26 h	[54]

Feed solutions	Membrane types	Enhanced methods	Feed temperature (°C)	Permeate temperature (°C)	Initial flux (L m^{-2} h^{-1})	Final flux (L m^{-2} h^{-1})	Initial conductivity (μs cm^{-1})	Final conductivity (μs cm^{-1})	Initial salt rejection (%)	Final salt rejection (%)	Ref.
SDS 35 g L^{-1} NaCl and 0.1~0.4 mM SDS	PVDF-HFP ENM	Fluorination Attaching nanomaterials-Fluorination	60	20	21.3	21.3@4 h	/	/	100	100@4 h	[134]
1 M NaCl and 0.1 mM SDS	PVDF-HFP ENM	Attaching nanomaterials-Fluorination	60	20	22.4	21.3@80 h	1.5	3.4@80 h	100	>99.9@80 h	[133]
1 M NaCl and 0.05~0.2 mM SDS	Quartz fiber membrane	Attaching nanomaterials-Fluorination	60	20	19.0	17.1@8 h	/	/	100	100@8 h	[158]
1 M NaCl and 0.1~0.3 mM SDS	PVDF-HFP ENM	Attaching nanomaterials-Fluorination	60	20	8	8@20 h	/	/	100	100@20 h	[132]
1 M NaCl and 0.1~0.3 mM SDS	Glass fiber membrane	Attaching nanomaterials-Fluorination	60	20	20.1	20.1@8 h	/	/	100	100@8 h	[157]
1 M NaCl and 0.3 mM SDS	Glass fiber membrane	Attaching nanomaterials-Fluorination	60	20	12.5	11.9@8 h	/	/	100	~100@8 h	[156]

Feed solutions	Membrane types	Enhanced methods	Feed temperature (℃)	Permeate temperature (℃)	Initial flux (L m^{-2} h^{-1})	Final flux (L m^{-2} h^{-1})	Initial conductivity (μs cm^{-1})	Final conductivity (μs cm^{-1})	Initial salt rejection (%)	Final salt rejection (%)	Ref.
1 M NaCl and 0.1~0.5 mM SDS	PVDF FSM	Attaching nanomaterials-Fluorination	60	20	18	10.26@8 h	/	/	100	100@8 h	[29]
1 M NaCl and 0.1~1.0 mM SDS	PVDF FSM	Attaching nanomaterials-Fluorination	60	20	16.4	13.7@22 h	/	/	100	~100@22 h	[162]
1 M NaCl and 2.0 mM SDS	Alumina HFM	Attaching nanomaterials-Fluorination	70	15	14.4	13.8@24 h	/	/	100	>99.9@24 h	[165]
35 g L^{-1} NaCl, 50 mg L^{-1} HA, and 0.1~0.4 mM SDS	PVDF FSM	Attaching nanomaterials-Fluorination	65	15	36.3	36.3@16 h	0	0@16 h	/	/	[31]
35 g L^{-1} NaCl, 1.26 g L^{-1} $CaCl_2$, 10 mg L^{-1} HA, and 10 mg L^{-1} SDBS	PVDF FSM	Attaching nanomaterials-Fluorination	70	20	35	35@150 h	1.56	5.12@150 h	/	/	[44]
100 g L^{-1} NaCl, 30 g L^{-1} $MgSO_4$, 1.26 g L^{-1} $CaCl_2$, 10	PVDF FSM	Attaching nanomaterials-Fluorination	70	20	37	33.85@90 h	1.56	6.68@90 h	/	/	[44]

Feed solutions	Membrane types	Enhanced methods	Feed temperature (°C)	Permeate temperature (°C)	Initial flux (L m^{-2} h^{-1})	Final flux (L m^{-2} h^{-1})	Initial conductivity (μs cm^{-1})	Final conductivity (μs cm^{-1})	Initial salt rejection (%)	Final salt rejection (%)	Ref.
mg L^{-1} HA, and 10 mg L^{-1} SDBS											
3.5% NaCl and 0.015% v/v oil	PVDF FSM	Attaching nanomaterials-Fluorination	60	20	24.29	22.15@48 h	/	/	100	99.4@48 h	[22]
3.5 wt % NaCl and 0.001%~0.01% v/v mineral oil	PVDF FSM	Attaching nanomaterials-Fluorination	70	20	28	~27@6 h	/	/	100	100@6 h	[17]
3.5 wt % NaCl and 0.001%~0.01% v/v kerosene	PVDF FSM	Attaching nanomaterials-Fluorination	70	20	28	~27@6 h	/	/	100	100@6 h	[17]
3.5 wt% NaCl and 200 ppm mineral oil	PVDF-HFP ENM	Attaching nanomaterials-Fluorination	60	20	19.11	16@72 h	/	/	100	~100@72 h	[131]
35 g L^{-1} NaCl and 80~320 mg L^{-1} oil	PVDF-HFP ENM	Attaching nanomaterials-Fluorination	60	20	21.3	21.3@4 h	/	/	100	~100@4 h	[134]
0.5 mM SDBS and 200 ppm hexadecane	PES FSM	Attaching nanomaterials-Fluorination	50	20	14.5	12.1@9.3 h	1.2	1.2@9.3 h	100	99.99@9.3 h	[145]

Feed solutions	Membrane types	Enhanced methods	Feed temperature (℃)	Permeate temperature (℃)	Initial flux (L m^{-2} h^{-1})	Final flux (L m^{-2} h^{-1})	Initial conductivity (μs cm^{-1})	Final conductivity (μs cm^{-1})	Initial salt rejection (%)	Final salt rejection (%)	Ref.
3.5 wt% NaCl and 150 ppm DTAB stabilized mineral oil	PVDF ENM	Attaching nanomaterials-Fluorination	60	20	21.5	20@12 h	1.74	2.4@12 h	~99.99	~99.99@12 h	[79]
1 M NaCl, 0.05 mM SDS, and 50~400 ppm oil	PVDF FSM	Attaching nanomaterials-Fluorination	60	20	16.4	13.3@22 h	/	/	100	100@22 h	[162]
1 M NaCl, 0.03 g L^{-1} TWEEN[j)], and 0.5 g L^{-1} crude oil	Quartz fiber membrane	Attaching nanomaterials-Fluorination	60	20	15.8	11.5@5.7 h	0	181@5.7 h	/	/	[158]
5 g L^{-1} NaCl, 0.5 mM SDBS, and 200 ppm hexadecane	PES FSM	Attaching nanomaterials-Fluorination	50	20	8.3	8.5@9.3 h	1.9	29.5@9.3 h	100	99.9@9.3 h	[145]
Mimetic AOM contaminated Seawater	PVDF-HFP ENM	Attaching nanomaterials-Fluorination	65	18	34.17	26.65@24 h	0	2.6@24 h	100	99.99@24 h	[52]
Seawater (East China Sea)	PVDF-HFP ENM	Attaching nanomaterials-Fluorination	60	20	35	34.86@50 h	0	0.6@50 h	/	/	[164]
Biologically	PVDF	Attaching	60	20	18.0	15.3@120 h	9.5	13@120 h	/	/	[41]

Feed solutions	Membrane types	Enhanced methods	Feed temperature (℃)	Permeate temperature (℃)	Initial flux (L m^{-2} h^{-1})	Final flux (L m^{-2} h^{-1})	Initial conductivity (μs cm^{-1})	Final conductivity (μs cm^{-1})	Initial salt rejection (%)	Final salt rejection (%)	Ref.
pre-treated coking wastewater	FSM	nanomaterials-Fluorination			18.3	17.2@120 h	9.5	8.3@120 h			
Synthetic shale gas wastewater	PVDF<br>FSM	Attaching nanomaterials-Fluorination	60	20	21.4<br>23.9	9.38@20 h<br>17.64@17.5 h	0<br>0	575@20 h<br>250@17.5 h	/	/	[54]
Imitative oil/gas production waste emulsion	PVDF<br>FSM	Attaching nanomaterials-Fluorination	60	20	4	2@16.5 h	31.96	207.3@16.5 h	/	/	[25]

a) Feed temperature: mostly 60 ℃; permeate temperature: mostly 20 ℃; all operation durations were the flux-stable time; the Character after @ is the operation duration.

b) HA: humic acid.

c) SDS: sodium dodecyl sulfate.

d) CTAB: cetyl trimethyl ammonium bromide.

e) SLS: sodium lauryl sulfonate.

f) DTAC: dodecyl trimethyl ammonium chloride.

g) DTAB: dodecyl trimethyl ammonium bromide.

h) SDBS: sodium dodecyl benzene sulfonate.

i) AOM: algal organic matter, which was prepared by mixing HA sodium salt, sodium-alginate (SA) sodium salt, and bovine serum albumin (BSA).[1]

j) TWEEN: polyoxyethylenesorbitan monolaurate.

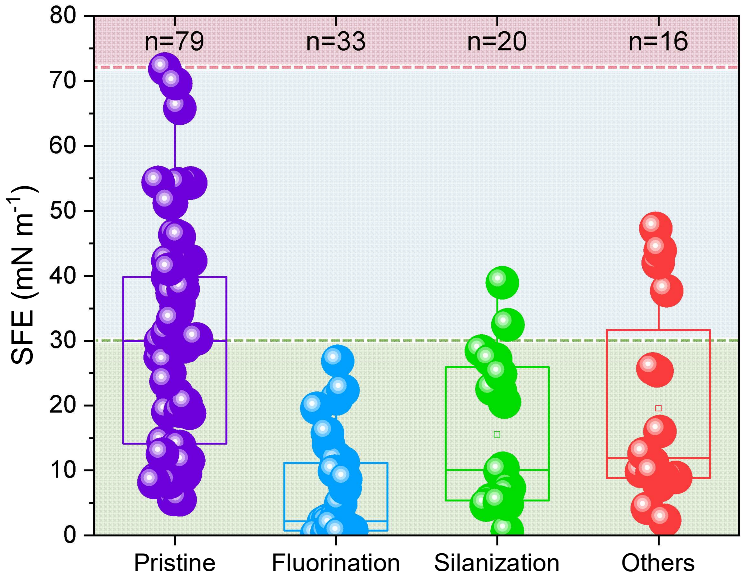

Figure S1. SFE values of pristine, fluorinated, silanized, and other modified membranes (n is the sample number). The red dashed line shows the surface tension of water (~72 mN m^{-1}),[166] and the green dashed line indicates the average SFE value of the pristine membrane (~30 mN m^{-1}). Raw data are listed in **Table S1**, Supporting Information.

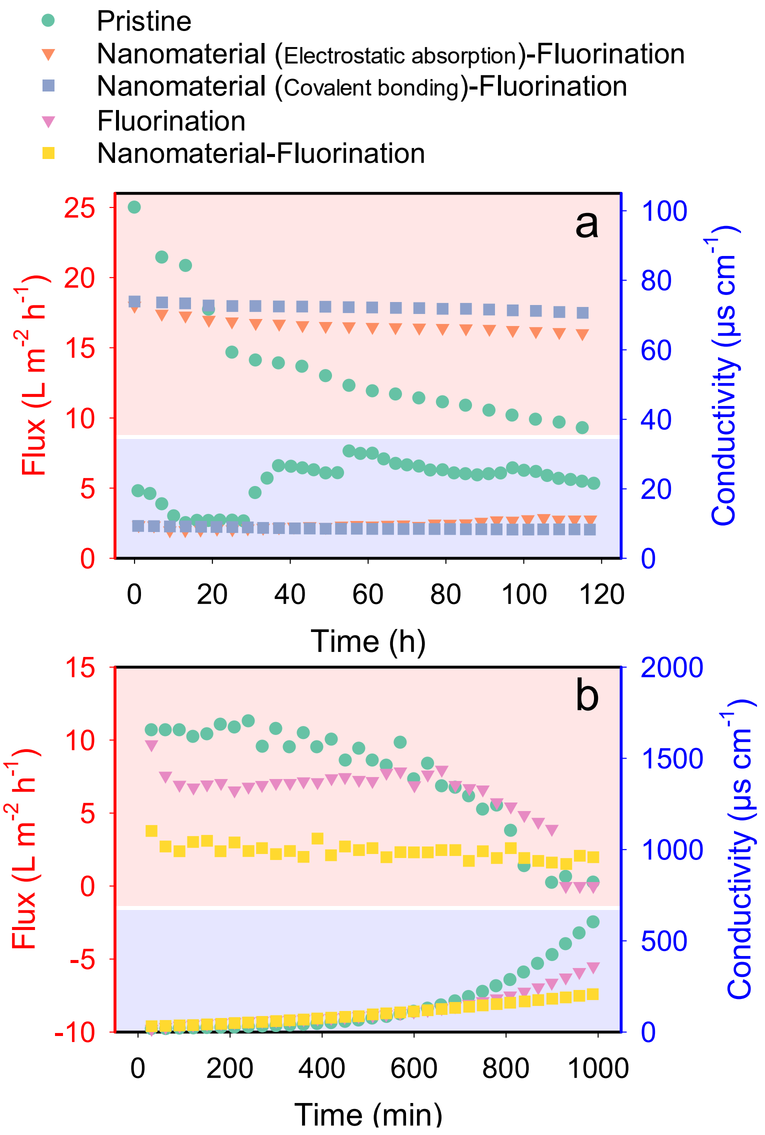

Figure S2. a) The permeate flux and conductivity of MD equipped with the pristine, attaching nanomaterials (electrostatic absorption)-fluorination, and attaching nanomaterials (covalent bonding)-fluorination membranes during the DCMD process. Experimental conditions: feed: the biologically pre-treated coking wastewater (COD = 127 ± 6 mg L^{-1}, DOC = 41.2 ± 1.2 mg L^{-1}, conductivity = 2400 ± 230 μs cm^{-1}), feed side temperature: 60 °C, permeate side temperature: 20 °C. Reproduced with permission.[41] Copyright 2019, Elsevier. b) The permeate flux and conductivity of MD equipped with the pristine, fluorination, and attaching nanomaterials-fluorination membranes during the DCMD process. Experimental conditions: feed: imitative oil/gas production waste emulsion, feed side temperature: 60 °C, permeate side temperature: 20 °C. Reproduced with permission.[25] Copyright 2018, Elsevier. The pristine membranes are PVDF flat-sheet membranes. The salmon pink and pinkish blue areas enclose the flux and conductivity data, respectively.

References

[1] B. J. Deka, E.-J. Lee, J. Guo, J. Kharraz, A. K. An, *Environ. Sci. Technol.* **2019**, 53, 4948.

[2] W. U. Rehman, A. Muhammad, M. Younas, C. Wu, Y. Hu, J. Li, *J. Membr. Sci.* **2019**, 584, 66.

[3] B. Xie, G. Xu, Y. Jia, L. Gu, Q. Wang, N. Mushtaq, B. Cheng, Y. Hu, *J. Membr. Sci.* **2021**, 625, 118978.

[4] I. Khan Swati, Q. Sohaib, H. Khan, M. Younas, A. Hosseini Monjezi, J. Li, M. Rezakazemi, *J. Mol. Liq.* **2022**, 351, 118566.

[5] V. M.-W. Yim, A. S.-W. Lo, B. J. Deka, J. Guo, J. A. Kharraz, I. T. Horváth, A. K. An, *Green Chem.* **2020**, 22, 3283.

[6] M. K. Alsebaeai, A. L. Ahmad, *J. Ind. Eng. Chem.* **2020**, 86, 13.

[7] A. A. Khan, M. I. Siyal, C.-K. Lee, C. Park, J.-O. Kim, *Sep. Purif. Technol.* **2019**, 210, 20.

[8] C. Adiche, K. Sundmacher, *Chem. Eng. Process. Process Intensification* **2010**, 49, 425.

[9] Z. Li, Y. Peng, Y. Dong, H. Fan, P. Chen, L. Qiu, Q. Jiang, *Appl. Surf. Sci.* **2014**, 317, 338.

[10] M. Wang, G. Liu, H. Yu, S.-H. Lee, L. Wang, J. Zheng, T. Wang, Y. Yun, J. K. Lee, *ACS Appl. Mater. Interfaces* **2018**, 10, 13452.

[11] J. Kujawa, E. Guillen-Burrieza, H. A. Arafat, M. Kurzawa, A. Wolan, W. Kujawski, *Food Bioproc. Tech.* **2015**, 8, 2146.

[12] Z. Xiao, R. Zheng, Y. Liu, H. He, X. Yuan, Y. Ji, D. Li, H. Yin, Y. Zhang, X.-M. Li, T. He, *Water Res.* **2019**, 155, 152.

[13] C. Liu, L. Chen, L. Zhu, *Water Res.* **2017**, 119, 33.

[14] D. Hou, C. Ding, C. Fu, D. Wang, C. Zhao, J. Wang, *Desalination* **2019**, 468, 114068.

[15] D. Hou, Z. Yuan, M. Tang, K. Wang, J. Wang, *J. Membr. Sci.* **2020**, 595, 117495.

[16] W. Jia, J. A. Kharraz, P. J. Choi, J. Guo, B. J. Deka, A. K. An, *J. Membr. Sci.* **2020**, 597, 117638.

[17] X. Li, H. Shan, M. Cao, B. Li, *J. Membr. Sci.* **2019**, 589, 117262.

[18] Z. Xiao, Z. Li, H. Guo, Y. Liu, Y. Wang, H. Yin, X. Li, J. Song, L. D. Nghiem, T. He, *Desalination* **2019**, 466, 36.

[19] T. A. Agbaje, S. Al-Gharabli, M. O. Mavukkandy, J. Kujawa, H. A. Arafat, *Desalination* **2018**, 436, 69.

[20] Z. Xiao, H. Guo, H. He, Y. Liu, X. Li, Y. Zhang, H. Yin, A. V. Volkov, T. He, *J. Membr. Sci.* **2020**, 599, 117819.

[21] S. Al-Gharabli, Z. Abu El-Rub, E. M. Hamad, W. Kujawski, Z. Flanc, K. Pianka, W. Jankowski, J. Kujawa, *Appl. Surf. Sci.* **2022**, 602, 154341.

[22] B. J. Deka, J. Guo, N. K. Khanzada, A. K. An, *Water Res.* **2019**, 165, 114982.

[23] A. Razmjou, E. Arifin, G. Dong, J. Mansouri, V. Chen, *J. Membr. Sci.* **2012**, 415-416, 850.

[24] W. Lee, Y. Choi, *Chem. Eng. J.* **2022**, 442, 136112.

[25] R. Zheng, Y. Chen, J. Wang, J. Song, X.-M. Li, T. He, *J. Membr. Sci.* **2018**, 555, 197.

[26]T. Arumugham, N. J. Kaleekkal, D. Rana, K. I. Sathiyanarayanan, *RSC Adv.* **2019**, 9, 41462.

[27] J. A. Kharraz, A. K. An, *J. Membr. Sci.* **2020**, 595, 117596.

[28] E. M.Hamad, S. Al-Gharabli, J. Kujawa, *Sep. Purif. Technol.* **2022**, 291, 120935.

[29] X. Du, M. Alipanahrostami, W. Wang, T. Tong, *ACS Appl. Mater. Interfaces* **2022**, 14, 23808.

[30] W. Zhang, B. Hu, Z. Wang, B. Li, *J. Membr. Sci.* **2021**, 622, 119038.
[31] W. Zhang, Z. Wang, B. Li, *J. Membr. Sci.* **2021**, 640, 119824.
[32] Z. Wei, Y. Jin, J. Li, L. Jia, Y. Ma, M. Chen, *Desalination* **2022**, 529, 115649.
[33] G. Zuo, R. Wang, *J. Membr. Sci.* **2013**, 447, 26.
[34] D. Hou, K. S. S. Christie, K. Wang, M. Tang, D. Wang, J. Wang, *J. Membr. Sci.* **2020**, 599, 117708.
[35] a)D. Rice, S. J. Ghadimi, A. C. Barrios, S. Henry, W. S. Walker, Q. Li, F. Perreault, *Environ. Sci. Technol.* **2020**, 54, 2548; b)H. Chang, B. Liu, Z. Zhang, R. Pawar, Z. Yan, J. C. Crittenden, R. D. Vidic, *Environ. Sci. Technol.* **2021**, 55, 1395.
[36]Y.-R. Chen, R. Xin, X. Huang, K. Zuo, K.-L. Tung, Q. Li, *J. Membr. Sci.* **2021**, 620, 118913.
[37] J. Kujawa, M. Zięba, W. Zięba, S. Al-Gharabli, W. Kujawski, A. P. Terzyk, *Chem. Eng. J.* **2022**, 438, 135574.
[38] J. Wang, L. Zheng, Z. Wu, Y. Zhang, X. Zhang, *J. Membr. Sci.* **2016**, 497, 183.
[39] E.-J. Lee, B. J. Deka, A. K. An, *J. Membr. Sci.* **2019**, 573, 570.
[40] S. Al-Gharabli, B. Al-Omari, W. Kujawski, J. Kujawa, *ACS Appl. Mater. Interfaces* **2021**, 13, 11268.
[41] J. Li, S. Guo, Z. Xu, J. Li, Z. Pan, Z. Du, F. Cheng, *J. Membr. Sci.* **2019**, 574, 349.
[42] J. Szczerbińska, W. Kujawski, J. M. Arszyńska, J. Kujawa, *J. Membr. Sci.* **2017**, 538, 1.
[43] L. Meng, J. Mansouri, X. Li, J. Liang, M. Huang, Y. Lv, Z. Wang, V. Chen, *J. Membr. Sci.* **2022**, 647, 120267.
[44] W. Zhang, Y. Lu, J. Liu, X. Li, B. Li, S. Wang, *J. Membr. Sci.* **2020**, 595, 117563.
[45] C. Boo, J. Lee, M. Elimelech, *Environ. Sci. Technol.* **2016**, 50, 12275.
[46] V. Karanikola, C. Boo, J. Rolf, M. Elimelech, *Environ. Sci. Technol.* **2018**, 52, 14362.
[47] X. Lu, Y. Peng, L. Ge, R. Lin, Z. Zhu, S. Liu, *J. Membr. Sci.* **2016**, 505, 61.
[48] X. Lu, Y. Peng, H. Qiu, X. Liu, L. Ge, *Desalination* **2017**, 413, 127.
[49] J. Lin, J. Du, S. Xie, F. Yu, S. Fang, Z. Yan, X. Lin, D. Zou, M. Xie, W. Ye, *Desalination* **2022**, 538, 115925.
[50] L. Zhong, L. An, Y. Han, Z. Zhu, D. Liu, D. Liu, D. Zuo, W. Wang, J. Ma, *Environ. Sci. Technol.* **2021**, 55, 11308.
[51] W. Wang, X. Du, H. Vahabi, S. Zhao, Y. Yin, A. K. Kota, T. Tong, *Nat. Commun.* **2019**, 10, 3220.
[52] J. Guo, B. J. Deka, K.-J. Kim, A. K. An, *Desalination* **2019**, 468, 114054.
[53] P. Jacob, T. Zhang, S. Laborie, C. Cabassud, *Desalination* **2019**, 468, 114086.
[54] H. Li, H. Feng, M. Li, X. Zhang, *J. Membr. Sci.* **2022**, 644, 120124.
[55] S. Al-Gharabli, Z. Flanc, K. Pianka, A. P. Terzyk, W. Kujawski, J. Kujawa, *Chem. Eng. J.* **2023**, 452, 139281.
[56] P. Jacob, S. Laborie, C. Cabassud, *Desalination* **2018**, 443, 307.
[57] B. Li, D. Hou, C. Li, Y. Yun, *Sep. Purif. Technol.* **2022**, 294, 121163.
[58] D. Feng, X. Li, Z. Wang, *J. Membr. Sci.* **2022**, 660, 120858.
[59] Z. Chen, D. Rana, T. Matsuura, D. Meng, C. Q. Lan, *Chem. Eng. J.* **2015**, 276, 174.
[60] L. Eykens, K. De Sitter, C. Dotremont, L. Pinoy, B. Van der Bruggen, *Desalination* **2016**, 392, 63.
[61]G. Racz, S. Kerker, Z. Kovács, G. Vatai, M. Ebrahimi, P. Czermak, *Period. Polytech-Chem.* **2014**, 58, 81.

[62] Z. Chen, D. Rana, T. Matsuura, Y. Yang, C. Q. Lan, *Sep. Purif. Technol.* **2014**, 133, 303.
[63] C. Boo, S. Hong, M. Elimelech, *Environ. Sci. Technol.* **2018**, 52, 14198.
[64] X. Liao, S. Chou, C. Gu, X. Zhang, M. Shi, X. You, Y. Liao, A. G. Razaqpur, *J. Membr. Sci.* **2023**, 665, 121130.
[65] N. Khumalo, L. Nthunya, S. Derese, M. Motsa, A. Verliefde, A. Kuvarega, B. B. Mamba, S. Mhlanga, D. S. Dlamini, *Sep. Purif. Technol.* **2019**, 211, 610.
[66] N. Hamzah, C. P. Leo, *Desalination* **2017**, 418, 79.
[67] H. K. Lee, S. S. Ray, D. T. Thanh Huyen, W. Kang, Y.-N. Kwon, *J. Membr. Sci.* **2021**, 629, 119280.
[68] K. J. Lu, J. Zuo, J. Chang, H. N. Kuan, T.-S. Chung, *Environ. Sci. Technol.* **2018**, 52, 4472.
[69] H. Cho, Y. Choi, S. Lee, *Desalination* **2018**, 437, 195.
[70] W. Shi, T. Li, M. Fan, H. Li, H. Zhang, X. Qin, *J. Ind. Eng. Chem.* **2022**, 108, 328.
[71] K. Li, D. Hou, C. Fu, K. Wang, J. Wang, *J. Environ. Sci. (China)* **2019**, 75, 277.
[72] M. Essalhi, M. Khayet, *J. Membr. Sci.* **2014**, 454, 133.
[73] M. Essalhi, M. Khayet, N. Ismail, O. Sundman, N. Tavajohi, *Desalination* **2021**, 510, 115086.
[74] G. Zheng, L. Yao, X. You, Y. Liao, R. Wang, J. J. Huang, *J. Membr. Sci.* **2021**, 620, 118918.
[75] C. Su, J. Chang, K. Tang, F. Gao, Y. Li, H. Cao, *Sep. Purif. Technol.* **2017**, 178, 279.
[76] P. Yadav, R. Farnood, V. Kumar, *Chemosphere* **2022**, 287, 132092.
[77] L.-F. Ren, J. Li, Y. Xu, J. Shao, Y. He, *Resour. Conserv. Recycl.* **2023**, 188, 106646.
[78] J. Li, L.-F. Ren, M. Huang, J. Yang, J. Shao, Y. He, *J. Membr. Sci.* **2022**, 650, 120404.
[79] X. Liao, Y. Wang, Y. Liao, X. You, L. Yao, A. G. Razaqpur, *J. Membr. Sci.* **2021**, 634, 119433.
[80] L. Zhong, Z. Zhu, Y. Han, Q. Wang, D. Liu, F. Cui, B. Li, W. Wang, *Environ. Sci. Nano* **2019**, 6, 2553.
[81] Y. Chen, K. J. Lu, T.-S. Chung, *J. Membr. Sci.* **2020**, 595, 117572.
[82] L. Deng, H. Ye, X. Li, P. Li, J. Zhang, X. Wang, M. Zhu, B. S. Hsiao, *Sep. Purif. Technol.* **2018**, 206, 14.
[83] Y. Chul Woo, Y. Chen, L. D. Tijing, S. Phuntsho, T. He, J.-S. Choi, S.-H. Kim, H. Kyong Shon, *J. Membr. Sci.* **2017**, 529, 234.
[84] C. Ji, Z. Zhu, L. Zhong, W. Zhang, W. Wang, *Desalination* **2021**, 519, 115185.
[85] L. N. Nthunya, L. Gutierrez, L. Lapeire, K. Verbeken, N. Zaouri, E. N. Nxumalo, B. B. Mamba, A. R. Verliefde, S. D. Mhlanga, *Sep. Purif. Technol.* **2019**, 228, 115793.
[86] Z. Li, B. Cheng, J. Ju, W. Kang, Y. Liu, *Desalination* **2021**, 501, 114834.
[87]X. Hu, X. Chen, M. Giagnorio, C. Wu, Y. Luo, C. Hélix-Nielsen, P. Yu, W. Zhang, *J. Membr. Sci.* **2022**, 661, 120850.
[88] M. Tang, D. Hou, C. Ding, K. Wang, D. Wang, J. Wang, *Sci. Total Environ.* **2019**, 696, 133883.
[89] W. Intrchom, S. Roy, S. Mitra, *Nanomaterials* **2020**, 10, 578.
[90] W. Jin, Y. Wu, H. Zhu, Y. Guo, T. Feng, C. Zhu, *Technol. Water Treat.* **2012**, 38, 23.
[91] Y. Chen, R. Zheng, J. Wang, Y. Liu, Y. Wang, X.-M. Li, T. He, *Desalination* **2017**, 424, 140.
[92] F. Heidarpour, J. Shi, S.-R. Chae, *Water Sci. Technol.* **2015**, 72, 908.
[93] H. Ramlow, R. A. F. Machado, A. C. K. Bierhalz, C. Marangoni, *Environ. Technol.* **2020**,

41, 2253.
[94] N. A. M. Ameen, S. S. Ibrahim, Q. F. Alsalhy, A. Figoli, *Water* **2020**, 12, 1575.
[95] B.-G. Im, J.-G. Lee, Y.-D. Kim, W.-S. Kim, *J. Membr. Sci.* **2018**, 565, 14.
[96] Y. Wang, H. Zhu, J. Li, Y. Guo, H. Zhang, J. Chen, *J. Funct. Mater.* **2015**, 46, 6070.
[97] M. M. A. Shirazi, A. Kargari, M. Tabatabaei, *Chem. Eng. Commun.* **2015**, 202, 457.
[98] M. Bhattacharya, S. K. Dutta, J. Sikder, M. K. Mandal, *J. Membr. Sci.* **2014**, 450, 447.
[99] C. D. Venzke, D. U. Rizzana, A. Giacobbo, M. A. S. Rodrigues, T. He, A. M. Bernardes, *J. Water Process. Eng.* **2021**, 39, 101722.
[100] A. Venault, Y. Chang, H.-H. Hsu, J.-F. Jhong, H.-S. Yang, T.-C. Wei, K.-L. Tung, A. Higuchi, J. Huang, *J. Membr. Sci.* **2013**, 439, 48.
[101] T. Jiříček, M. Komárek, J. Chaloupek, T. Lederer, *J. Nanomater.* **2016**, 2016, 9327431.
[102] H. Wu, F. Shen, J. Wang, Y. Wan, *J. Membr. Sci.* **2018**, 550, 436.
[103] J. Kujawa, E. Chrzanowska, W. Kujawski, *Chem. Zvesti.* **2019**, 73, 565.
[104] L. Francis, N. Ghaffour, A. S. Alsaadi, S. P. Nunes, G. L. Amy, *J. Membr. Sci.* **2014**, 455, 103.
[105] H. Zhang, M. Liu, D. Sun, B. Li, P. Li, *Chem. Eng. Process. Process Intensification* **2016**, 110, 52.
[106] S. Jeong, S. Lee, H.-T. Chon, S. Lee, *Desalination* **2014**, 349, 115.
[107] C. Yue, H. Zhu, F. Wang, Y. Guo, H. Zhang, J. Chen, *Technol. Water Treat.* **2014**, 40, 43.
[108] M. M. A. Shirazi, A. Kargari, M. Tabatabaei, *Chem. Eng. Process. Process Intensification* **2014**, 76, 16.
[109] S. Kim, D. E. Heath, S. E. Kentish, *Adv. Mater. Interfaces* **2022**, 9, 2200786.
[110] M. Xu, J. Cheng, X. Du, Q. Guo, Y. Huang, Q. Huang, *J. Membr. Sci.* **2022**, 641, 119876.
[111] A. Yadav, K. Singh, V. K. Shahi, *Desalination* **2022**, 525, 115501.
[112] M. M. Aljumaily, H. M. Alayan, A. A. Mohammed, M. A. Alsaadi, Q. F. Alsalhy, A. Figoli, A. Criscuoli, *Appl. Water Sci.* **2022**, 12, 28.
[113] A. Yadav, K. Singh, A. B. Panda, P. K. Labhasetwar, V. K. Shahi, *J. Water Process. Eng.* **2021**, 44, 102393.
[114] M. J. Toh, P. C. Oh, M. I. S. Mohd Shaufi, *IOP Conference Series: Materials Science and Engineering* **2020**, 778, 012176.
[115] X. Wang, C. Xiao, H. Liu, Q. Huang, J. Hao, H. Fu, *Materials* **2018**, 11, 443.
[116] X. Wang, C. Xiao, H. Liu, M. Chen, J. Hao, Y. Wu, *RSC Adv.* **2018**, 8, 27754.
[117] A. Yadav, R. V. Patel, P. K. Labhasetwar, V. K. Shahi, *J. Water Process. Eng.* **2021**, 43, 102317.
[118] S. S. Ray, C. K. Deb, H.-M. Chang, S.-S. Chen, M. Ganesapillai, *J. Appl. Polym. Sci.* **2019**, 136, 48021.
[119] a)M. M. Aljumaily, M. A. Alsaadi, N. A. Hashim, Q. F. Alsalhy, F. S. Mjalli, M. A. Atieh, A. Al-Harrasi, *Chem. Eng. Res. Des.* **2018**, 138, 248; b)L. Xie, X. Huang, K. Yang, S. Li, P. Jiang, *J. Mater. Chem. A* **2014**, 2, 5244.
[120] M. J. Toh, P. C. Oh, A. L. Ahmad, J. Caille, *Korean J. Chem. Eng.* **2019**, 36, 1854.
[121] S. Fadhil, Q. F. Alsalhy, H. F. Makki, R. Ruby-Figueroa, T. Marino, A. Criscuoli, F. Macedonio, L. Giorno, E. Drioli, A. Figoli, *Chem. Eng. Commun.* **2019**, 206, 237.

[122] Q. F. Alsalhy, S. S. Ibrahim, F. A. Hashim, *Chem. Eng. Res. Des.* **2018**, 130, 95.
[123] I. Tournis, D. Tsiourvas, Z. Sideratou, L. G. Boutsika, A. Papavasiliou, N. K. Boukos, A. A. Sapalidis, *Environ. Sci. Water Res. Technol.* **2022**, 8, 2373.
[124] M. J. Toh, P. C. Oh, T. L. Chew, A. L. Ahmad, *Sep. Purif. Technol.* **2020**, 244, 116543.
[125] A. Dastbaz, J. Karimi-Sabet, H. Ahadi, Y. Amini, *Desalination* **2017**, 424, 62.
[126] J. Song, Q. Deng, M. Huang, Z. Kong, *Environ. Res.* **2022**, 204, 111892.
[127] G. Tan, D. Xu, Z. Zhu, X. Zhang, J. Li, *J. Membr. Sci.* **2022**, 658, 120751.
[128] C. Su, T. Horseman, H. Cao, K. Christie, Y. Li, S. Lin, *Environ. Sci. Technol.* **2019**, 53, 11801.
[129] Y. C. Woo, M. Yao, W.-G. Shim, Y. Kim, L. D. Tijing, B. Jung, S.-H. Kim, H. K. Shon, *J. Membr. Sci.* **2021**, 623, 119028.
[130] Z. Chen, J. Li, J. Zhou, X. Chen, *Chem. Eng. J.* **2023**, 451, 138473.
[131] Y. Xu, Y. Yang, X. Fan, Z. Liu, Y. Song, Y. Wang, P. Tao, C. Song, M. Shao, *Desalination* **2021**, 499, 114832.
[132] J. Lee, C. Boo, W.-H. Ryu, A. D. Taylor, M. Elimelech, *ACS Appl. Mater. Interfaces* **2016**, 8, 11154.
[133] B. J. Deka, J. Guo, A. K. An, *J. Membr. Sci.* **2021**, 624, 119089.
[134] X. Li, W. Qing, Y. Wu, S. Shao, L. E. Peng, Y. Yang, P. Wang, F. Liu, C. Y. Tang, *ACS Appl. Mater. Interfaces* **2019**, 11, 47963.
[135] J. Guo, B. J. Deka, P. W. Wong, J. Sun, A. K. An, *Desalination* **2021**, 520, 115314.
[136] X.-Q. Wu, X. Wu, T.-Y. Wang, L. Zhao, Y. B. Truong, D. Ng, Y.-M. Zheng, Z. Xie, *J. Membr. Sci.* **2020**, 606, 118075.
[137] X. An, Y. Bai, G. Xu, B. Xie, Y. Hu, *Desalination* **2020**, 477, 114264.
[138] W. Jia, J. A. Kharraz, J. Guo, A. K. An, *J. Membr. Sci.* **2020**, 611, 118360.
[139] L. Zhou, C. L. Li, P. T. Chang, S. H. Tan, A. L. Ahmad, S. C. Low, *Desalination* **2022**, 527, 115594.
[140] X. Wen, F. Li, B. Jiang, X. Zhang, X. Zhao, *J. Chem. Technol. Biotechnol.* **2018**, 93, 2252.
[141] M. Rezaei, W. Samhaber, *Chem. Eng. Trans.* **2016**, 47, 373.
[142] A. Boubakri, A. Hafiane, S. A. T. Bouguecha, *Arab. J. Chem.* **2017**, 10, S3475.
[143] a)Y. Shao, M. Han, Y. Wang, G. Li, W. Xiao, X. Li, X. Wu, X. Ruan, X. Yan, G. He, X. Jiang, *J. Membr. Sci.* **2019**, 579, 240; b)Y. Wang, G. He, Y. Shao, D. Zhang, X. Ruan, W. Xiao, X. Li, X. Wu, X. Jiang, *Sep. Purif. Technol.* **2019**, 214, 11.
[144] L. Eykens, K. De Sitter, C. Dotremont, W. De Schepper, L. Pinoy, B. Van Der Bruggen, *Appl. Sci.* **2017**, 7, 118.
[145] A. A. Khan, M. I. Siyal, J.-O. Kim, *Chemosphere* **2021**, 263, 128140.
[146] J. Zuo, S. Bonyadi, T.-S. Chung, *J. Membr. Sci.* **2016**, 497, 239.
[147] J. Quan, J. Yu, Y. Wang, Z. Hu, *J. Membr. Sci.* **2022**, 648, 120353.
[148] H. Fan, A. Gao, G. Zhang, S. Zhao, J. Cui, Y. Yan, *J. Membr. Sci.* **2020**, 602, 117933.
[149] M. A. Ajdar, A. Azdarpour, A. Mansourizadeh, B. Honarvar, *Polym. Test.* **2019**, 76, 1.
[150] C. Li, X. Li, X. Du, Y. Zhang, W. Wang, T. Tong, A. K. Kota, J. Lee, *Environ. Sci. Technol.* **2020**, 54, 10333.
[151] M.-Y. Yang, J.-W. Wang, L. Li, B.-B. Dong, X. Xin, S. Agathopoulos, *J. Eur. Ceram. Soc.* **2019**, 39, 442.

[152] J. Kujawa, A. Rozicka, S. Cerneaux, W. Kujawski, *Colloids Surf. A Physicochem. Eng. Asp.* **2014**, 443, 567.

[153] S. A. Alftessi, M. H. D. Othman, M. R. Adam, T. M. Farag, A. Mustafa, T. Matsuura, J. Jaafar, M. A. Rahman, A. F. Ismail, *Desalination* **2022**, 532, 115705.

[154] M. H. Abd Aziz, M. H. Dzarfan Othman, N. H. Alias, T. Nakayama, Y. Shingaya, N. A. Hashim, T. A. Kurniawan, T. Matsuura, M. A. Rahman, J. Jaafar, *J. Membr. Sci.* **2020**, 607, 118137.

[155] X. Chen, X. Gao, K. Fu, M. Qiu, F. Xiong, D. Ding, Z. Cui, Z. Wang, Y. Fan, E. Drioli, *Desalination* **2018**, 443, 212.

[156] L.-H. Chen, A. Huang, Y.-R. Chen, C.-H. Chen, C.-C. Hsu, F.-Y. Tsai, K.-L. Tung, *Desalination* **2018**, 428, 255.

[157] C. Boo, J. Lee, M. Elimelech, *Environ. Sci. Technol.* **2016**, 50, 8112.

[158] C. Li, X. Li, X. Du, T. Tong, T. Y. Cath, J. Lee, *ACS Appl. Mater. Interfaces* **2019**, 11, 18456.

[159] J. Kujawa, S. Al-Gharabli, W. Kujawski, K. Knozowska, *ACS Appl. Mater. Interfaces* **2017**, 9, 6571.

[160] J. A. Arnot, F. A. P. C. Gobas, *Environ. Rev.* **2006**, 14, 257.

[161] R. Garg, C. J. Smith, *Food Chem. Toxicol.* **2014**, 69, 252.

[162] J. A. Kharraz, M. U. Farid, N. K. Khanzada, B. J. Deka, H. A. Arafat, A. K. An, *Water Res.* **2020**, 174, 115600.

[163] E.-J. Lee, B. J. Deka, J. Guo, Y. C. Woo, H. K. Shon, A. K. An, *Environ. Sci. Technol.* **2017**, 51, 10117.

[164] X. Xue, G. Tan, Z. Zhu, *ACS Appl. Mater. Interfaces* **2021**, 13, 45977.

[165] L.-H. Chen, Y.-R. Chen, A. Huang, C.-H. Chen, D.-Y. Su, C.-C. Hsu, F.-Y. Tsai, K.-L. Tung, *J. Membr. Sci.* **2018**, 564, 227.

[166] I. M. Hauner, A. Deblais, J. K. Beattie, H. Kellay, D. Bonn, *J. Phys. Chem. Lett.* **2017**, 8, 1599.
